# Topological Analysis of the Carbon-Concentrating CETCH Cycle and a Photorespiratory Bypass Reveals Boosted CO_2_-Sequestration by Plants

**DOI:** 10.3389/fbioe.2021.708417

**Published:** 2021-11-01

**Authors:** Özge Osmanoglu, Mariam Khaled AlSeiari, Hasa Abduljaleel AlKhoori, Shabana Shams, Elena Bencurova, Thomas Dandekar, Muhammad Naseem

**Affiliations:** ^1^ Department of Bioinformatics, Functional Genomics and Systems Biology Group, Biocenter, University of Würzburg, Am Hubland, Würzburg, Germany; ^2^ College of Natural and Health Sciences, Department of Life and Environmental Sciences, Zayed University, Abu Dhabi, UAE; ^3^ Department of Animal Sciences, Quaid-i-Azam University, Islamabad, Pakistan

**Keywords:** CO_2_-sequestration, photorespiration, elementary modes, synthetic pathways, carboxylation, metabolic modeling, CETCH cycle

## Abstract

Synthetically designed alternative photorespiratory pathways increase the biomass of tobacco and rice plants. Likewise, some *in planta*–tested synthetic carbon-concentrating cycles (CCCs) hold promise to increase plant biomass while diminishing atmospheric carbon dioxide burden. Taking these individual contributions into account, we hypothesize that the integration of bypasses and CCCs will further increase plant productivity. To test this *in silico*, we reconstructed a metabolic model by integrating photorespiration and photosynthesis with the synthetically designed alternative pathway 3 (AP3) enzymes and transporters. We calculated fluxes of the native plant system and those of AP3 combined with the inhibition of the glycolate/glycerate transporter by using the YANAsquare package. The activity values corresponding to each enzyme in photosynthesis, photorespiration, and for synthetically designed alternative pathways were estimated. Next, we modeled the effect of the crotonyl-CoA/ethylmalonyl-CoA/hydroxybutyryl-CoA cycle (CETCH), which is a set of natural and synthetically designed enzymes that fix CO₂ manifold more than the native Calvin–Benson–Bassham (CBB) cycle. We compared estimated fluxes across various pathways in the native model and under an introduced CETCH cycle. Moreover, we combined CETCH and AP3-w/plgg1RNAi, and calculated the fluxes. We anticipate higher carbon dioxide–harvesting potential in plants with an AP3 bypass and CETCH–AP3 combination. We discuss the *in vivo* implementation of these strategies for the improvement of C3 plants and in natural high carbon harvesters.

## Introduction

The concentration of CO₂ in the atmosphere is almost 418.30 parts per million (ppm-on April 15, 2021, https://www.esrl.noaa.gov/gmd/ccgg/trends/monthly.html), which represents a ∼47% increase since the beginning of the industrial revolution. The ever-increasing per capita CO₂ emissions are further limiting our options of mitigating the adverse effects of climate change. Biological processes such as photosynthesis and respiration are the two main attributes that reciprocally regulate net CO₂ concentrations in the atmosphere, but the anthropogenic emissions disturb the balance between CO₂ release and CO_2_ fixation by the Earth vegetation (Dusenge et al., 2019). Although higher CO₂ concentration in the atmosphere is always perceived as a problem, external CO₂ feedstock can be harnessed into an opportunity if the CO₂-sequestering capability of the land plants is enhanced (Wurtzel et al., 2019; Naseem et al., 2020). Whether or not the higher concentrations of CO₂ in the atmosphere can function as a selection cue on the Earth vegetation to enhance CO₂ sequestration as a mean of adaptation to global change is still not clear. However, C4 (plants produce 4-C compounds as their first stable product: spatial relocation of CO₂ fixation) and CAM (Crassulacean acid metabolism: temporal fixation of CO₂) plants have evolved CO_2_-fixation systems with a higher photosynthetic rate than C3 plants (Cousins et al., 2020; Ferrari et al., 2020). However, most plant species are C3 in nature and will need an evolutionary time scale to naturally evolve CO₂ hyper-sequestration rates than their current capacities. Synthetic evolution on the other hand deals with designing and realization of artificial metabolic pathways that can accelerate evolution with prospects for climate mitigation strategies and precision agriculture (Gleizer et al., 2019; Wurtzel et al., 2019; Kalluri et al., 2020; Miller et al., 2020). The rewiring and redesigning of plant carbon-assimilatory pathways such as the Calvin–Benson–Bassham (CBB) Cycle and the bypassing of plant photorespiration on alternative routes hold promise for higher plant biomass production (Bar-Even et al., 2010; Wurtzel et al., 2019; Naseem et al., 2020). In recent years, many *in vitro*– and *in vivo*–realized CO₂-fixing artificial cycles (Naseem et al., 2020) have been introduced. Two of these synthetically designed approaches got much attention in recent years: 1) bypassing of photorespiration (Kebeish et al., 2007; Xin et al., 2015; South et al., 2019; Naseem et al., 2020) and 2) carbon-concentrating mechanisms (CCMs) (Lin et al., 2014; Schwander et al., 2016; Miller et al., 2020; Naseem et al., 2020). Here, we investigate the topological feasibility of two such artificial cycles and their integration for hyper-carbon sequestration by land plants.

C3 plants such as wheat, rice, and soybeans lose 30–50% of their photosynthetic conversion efficiency by encouraging the oxygenation of ribulose-1,5-biphosphate (RuBP) by the enzyme ribulose-1,5-biphosphate carboxylase-oxygenase (Rubisco) (Bauwe et al., 2010; Dusenge et al., 2019). C4 and CAM plants have overcome these penalties by fixing CO₂ more efficiently prior to the onset of the CBB cycle (Xin et al., 2015; Dusenge et al., 2019). The potential benefits of modulating plant photorespiratory pathways into high biomass production plants have long been envisaged (Xin et al., 2015; Dusenge et al., 2019; Naseem et al., 2020). In this context, an increased *Arabidopsis* biomass through photorespiratory bypassing in the chloroplast has already been attempted and has thus paved the way for crop improvements (Kebeish et al., 2007). Following the principles of bypassing endogenous photorespiratory pathways, South et al. (South et al., 2019; Naseem et al., 2020) synthetically designed three glycolate metabolic routes (alternative photorespiration: AP1, AP2, and AP3) that were incorporated in tobacco plants. AP1 converts glycolate to glycerate using *E. coli* genes that encode for enzymes such as glycolate dehydrogenase, glyoxylate carboligase, and tartronic semi-aldehyde reductase (Kebeish et al., 2007; South et al., 2019; Naseem et al., 2020). Likewise, AP2 encompasses three introduced genes: glycolate oxidase, malate synthase, and catalase. AP3 relies on two different enzymes such as glycolate dehydrogenase (CrGDH) and malate synthase (MS) from *Chlamydomonas reinhardtii* and *Cucurbita maxima*, respectively (Maier et al., 2012 (designed first version); South et al., 2019). Besides the implementation of AP pathways, South et al. (2019) also inhibited the transportation of glycolate from the chloroplast to the native pathway by RNAi suppression of the glycolate/glycerate transporter 1 PLGG1 gene (South et al., 2019; Naseem et al., 2020). In comparison to the wild-type plants, the AP3-w/plgg1-RNAi plants manifested higher photosynthetic efficiency (ca. 40%), thereby leading to increased biomass production of tobacco plants under field conditions.

A completely different synthetic biology approach that can potentially increase the plant biomass production exploits the carbon-concentrating mechanisms (see Naseem et al., 2020 for a comparative overview). One promising development pertaining the efficient fixation of CO₂ is the *in vitro* implementation of the crotonyl-CoA/ethylmalonyl-CoA/hydroxybutyryl-CoA (CETCH) cycle (Schwander et al., 2016; Miller et al., 2020). This artificial cycle comprises 17 (16 natural enzymes from various species and one synthetically designed) naturally and synthetically acquired enzymes that convert CO₂ into organic molecules at a rate of 5 nmol CO₂ min-1 mg-1 of core CETCH proteins, whereas the natural CBB cycle fixes CO₂ with a rate of 1–3 nmol CO₂ min-1 mg-1 of the CBB proteins (Miller et al., 2020; Naseem et al., 2020). The CETCH cycle was established with enzymes originating from nine different organisms of all three kingdoms of life and optimized in several rounds by enzyme engineering and metabolic proofreading. The CETCH cycle encompasses only few ATP-using reactions and therefore requires less energy for its operation as compared to other aerobic CO₂-fixation pathways (Schwander et al., 2016; Miller et al., 2020; Naseem et al., 2020). One limitation of CETCH is the production of glyoxylate which is a comparatively less active metabolic intermediate and requires the presence of acetyl-CoA or propanoyl-CoA (South et al., 2019) when its conversion to other metabolites is required. Also, glyoxylate is not densely connected to other major metabolic pathways. Therefore, the *in vivo* realization of CETCH incorporation in plants would require special attention (Yang et al., 2019; Naseem et al., 2020). Despite these bottlenecks associated with any synthetically designed pathway, CETCH is still the most efficient artificial cycle that fixes several folds more CO₂ than the natural CBB does (Schwander et al., 2016; Miller et al., 2020; Naseem et al., 2020). We reconstructed models that harbor both AP3-bypasses and the CETCH cycle as well as their combination to envision metabolic dynamics across various downstream pathways.

Besides the artificial CETCH and AP3 pathways, there are also other CO_2_-harvesting alternatives (Naseem et al., 2020) which can be adopted for enhanced CO_2_ sequestration in plants. For instance, the GOC (glycolate oxidase–oxalate–catalase) bypass developed by Shen et al. (2019) is an interesting alternative to the AP3 bypass. GOC oxidizes glycolate completely to CO_2_ in the chloroplast. It would be interesting to see which of the bypasses (AP3 vs GOC) would be more energy efficient when it comes to their co-integration with the CETCH cycle. Likewise, MOG (malonyl-CoA-oxaloacetate-glyoxylate) cycles developed by Bar-Even et al. (2010) are alternative to the CETCH cycle. It is generally believed that MOG cycles use HCO_3_^-^ instead of CO_2_, and thus sequester CO_2_ in an indirect manner. A comparison in terms of energetics and efficiency between CETCH and MOG cycles would be very intriguing for the *in planta* realization of these various synthetic pathways.

To integrate these CO_2_ harvesting pathways into predictive models, we used structural metabolic network modeling. This is a common approach based on steady states in cellular metabolism. In contrast to dynamic metabolic modeling, the structural network modeling does not require knowledge of detailed kinetic parameters. With the structural approach, a metabolic model can be analyzed by knowing which enzymes are present and considering initial constraints. We used elementary mode analysis (EMA), a metabolic pathway analysis (MPA) method. EMA calculates the “minimal set of enzymes that can generate valid steady states” (overview in Supplementary text and Supplementary Figure S1, details in Schuster et al., 1999). In other words, elementary modes (EMs) are the non-decomposable paths in a metabolic network between an input and an output metabolite. Thus, elementary modes can neither be a combination of smaller modes nor they can be broken down into smaller modes that fulfill the steady-state condition (Schuster et al., 2000; Volkova et al., 2020). An alternative method is extreme pathway analysis (EPA), which looks for a subset of elementary pathways. The subset consists of the so-called extreme pathways as they include the minimal number of pathways to define the whole biochemical system by their linear combination (Liang et al., 2011).

Here, we provide extensive calculations on the effect of these pathways in models mimicking chloroplastic and peroxisomal connectivity. Our analysis of each model shows that 100% RNAi suppression of PLGG1 (plastidic glycolate/glycerate transporter 1) diminishes the photorespiratory flux while increasing photosynthetic enzyme activities in the chloroplast and thereby confirming the metabolic phenotype (South et al., 2019) associated with the AP3 bypass in plants. Although experimentally measured fluxes are still unknown for these modifications, our analysis nevertheless shows the resulting changes in terms of elementary modes and supports the finding that the AP3 bypass directs the flux of glycolate into malate, minimizing photorespiratory loss. Furthermore, we discuss that the biological feasibility of the CETCH cycle in combination with fully suppressed PLGG1 is lower than the biological feasibility of the CETCH cycle in combination with partially suppressed PLGG1 expression. The integration of AP3, CETCH, and their combination with and without the suppression of PLGG1 stimulate the CBB cycle activities to various degrees. We modeled the activity (flux distribution) of MS as readouts of the modified pathways such that CETCH co-integration with AP3 resulted in higher malate accumulation in comparison to AP3 integration alone. We thus advocate that the co-integration of AP3 and the CETCH cycle with partial suppression of the gylocolate transporter would offer boosted carbon sequestration possibility than that of native CBB cycles in plants. Taken together, our integrated topological metabolic network analysis has future implications for the realization of novel synthetic cycles with implications both for food security and climate mitigation strategies.

## Methods

### The Reconstruction of Plant Metabolic Models Harboring CO_2_ Sequestration Aided by Native Cycles and Synthetic Biology Pathways

The reconstruction of the native metabolic model was achieved by integrating different KEGG (Kanehisa and Goto, 2000) modules as well as manual curation. First, the CBB cycle was assembled by KEGG module M00165 which is complemented by two further reactions that are catalyzed by ribulose phosphate 3-epimerase and triose-phosphate isomerase (Schwander et al., 2016). The photorespiration module was assembled with the reactions from KEGG module M00532. For light-dependent reactions of photosynthesis, a manual inspection of the KEGG map00195 and MetaCyc (Caspi et al., 2020) PWY-101 was performed. Five reactions were included by making use of both sources, and these reactions are catalyzed by Photosystem II, Cytochrome b6f complex, Photosystem I, ferredoxin---NADP + reductase, and ATP synthase. It is noteworthy to mention that the required transporters linking metabolites among the chloroplast, mitochondria, and peroxisome were also manually added to the model. Reversibility to specific reactions was assigned from MetaCyc (Caspi et al., 2020). The complete list of reactions in the native metabolic model is given inSupplementary Table S1. The reactions for the photorespiration bypass pathway were adapted from alternative pathway 3 described in the study by South et al. (2019) and for the CETCH cycle described in the study by Schwander et al. (2016).

### Elementary Mode Analysis of the Native and Synthetic Metabolic Networks in Plants

Elementary modes (EM) are the non-decomposable paths in a metabolic network that can keep the network at a balanced state, in which internal metabolites are neither accumulated nor consumed, and their concentrations stay constant. In summation, they are groups of enzymes that can generate a steady state and cannot be broken down to smaller flux modes (overview in Supplementary text and Supplementary Figure S1, details in Schuster et al., 1999; Schuster et al., 2000).

A crucial step for the calculation of elementary modes is to define each metabolite as external or internal. Calculation of elementary flux modes follows paths of balanced internal metabolites between external metabolites as sources and drains (Schuster et al., 1999). External metabolites include inputs and outputs of the model and the pool of metabolites that participate in many reactions, and thus are buffered. Typical examples are nutrients or excreted products. Internal metabolites, on the other hand, are balanced metabolites in the system that are formed and consumed equally by the involved enzyme chain (overview in Supplementary text and Supplementary Figure S1, details in Schuster et al., 1999).

We applied the concept of internal or external metabolites on each and every model that we analyzed in our study. Metabolites that were either only formed or only consumed by one or more reactions were defined as external. Metabolites such as ADP, ATP, H₂O, CO₂, and H^+^ were considered external since they are relatively stable pools (Schwarz et al., 2007). They are also called “currency” metabolites as they are frequently exchanged in many reactions of the CETCH and AP3 integration models. To prevent combinatorial explosion, they are considered to be well buffered due to the many reactions that produce or consume them. In this type of pathway analysis, any external or pool metabolite is assumed to be available in sufficient quantity and need not be balanced. As we mentioned above, only the internal metabolites need to be balanced. Furthermore, we also defined metabolites that were involved in more than three reactions as external as they are well buffered. Therefore, only metabolites with a total of two or three connections in different directions (consumed and produced) were considered internal (Dandekar et al., 2003; Srivastava et al., 2019) in our model reconstruction process.

The calculation of elementary modes was performed in YANAsquare (Schwarz et al., 2007), implementing the Metatool algorithm (von Kamp and Schuster, 2006). Elementary mode analysis is performed as follows.

First flux modes are calculated by the following equation:

N*v = 0, (1)

where N is the stoichiometric matrix with a size of m (number of internal metabolites) x n (number of reactions) and v is the flux vector. All flux vectors that fulfill the steady-state criteria are defined by equation. Eq. 1 represents all possible paths (flux modes) through the metabolic model that satisfy the condition that all internal metabolites should be balanced. These flux modes are then defined as elementary if they are non-decomposable (Schwarz et al., 2005).

YANAsquare was also used to assign relative fluxes to each enzyme that represent the activity of the enzymes in each metabolic model. The calculation of enzyme activities is based on the number of EMs an enzyme is part of as well as its coefficients in the corresponding EMs. The enzyme activities represent the strength of the flux carried by each enzyme. By default, this flux distribution is calculated assuming that all EMs are equally and fully active. However, the activities of the EMs can be tuned based on experimental data on enzymes or metabolite concentrations. Nevertheless, without experimental data, the predicted enzyme activities are helpful for the identification of relevant enzymes that carry a higher flux in a given model. Moreover, higher enzyme activities reflect shifts in flux distribution caused by topological changes we introduce in the model (Schwarz et al., 2005; Kaltdorf et al., 2016).

## Results and Discussion

### Bypassing of Photorespiratory Pathways and the Introduction of Synthetic Cycles Generate Additional Elementary Modes in Plant Carbon Fixation Networks

**FIGURE 1:**
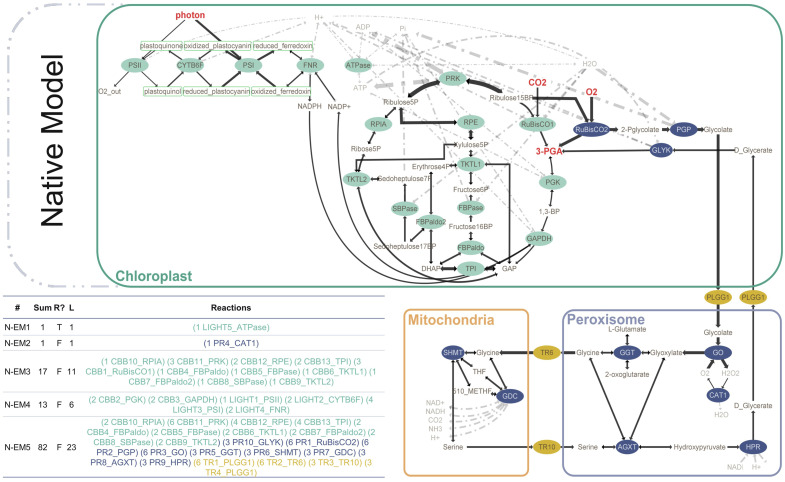
Native model of photosynthesis (green) and photorespiration (blue) with transporters between organelles (gold). The elementary modes are calculated according to reversibility and metabolite classification (internal/external). Reversible reactions are shown with double-sided arrows. Sum: flux sum, R: reversibility, L: length.

We first built the native model to represent the native state of photosynthesis and photorespiration. The native model (Figure 1) contains the light-dependent reactions (green), Calvin–Benson–Bassham (CBB) cycle (green), photorespiration (blue), and the relavant transporters in photorespiration (gold). The main inputs of the native model are CO₂ and O₂, while 3-phosphoglycerate (3-PGA) is designated as the main output of the model. Another output is glyceraldehyde 3-phosphate (GAP); however, we marked 3-PGA as our main output since it is the common product of CBB and photorespiration (Figure 1). The native model consists of 32 reactions (15 reversible and 17 irreversible) and 56 metabolites, in which 32 are internal (Supplementary Table S1 and Supplementary Table S2). Elementary mode analysis on the native model by YANAsquare resulted in five native elementary modes (N-EMs) (Figure 1). N-EM1 and N-EM2 consist of only one enzyme: ATPase in the chloroplast and catalase (CAT) in peroxisome. N-EM3 and N-EM4 span the photosynthetic reactions, which are split into two modes. N-EM3 covers most of the CBB cycle, resulting in the production of 3-PGA by consuming CO_2_, ATP, H_2_O, and GAP. It is then complemented by N-EM4 that represents the connection between the light-dependent reactions and the CBB cycle by the reducing agent NADPH. On the other hand, N-EM4 consumes 3-PGA and produces GAP by using ATP and NADPH produced by light-dependent reactions. N-EM5 overlaps with N-EM3 (especially in CBB cycle reactions); however, it represents the photorespiration in addition. It includes both the oxygenase and carboxylase functions of Rubisco. In this long elementary mode (Figure 1; Table 1), all reactions of photorespiration are included along with the transporters needed for the transportation of metabolites among cellular organelles such as chloroplast, peroxisome, and mitochondria (the net reactions for each EM is given in Supplementary TableS3).

**TABLE 1:** Elementary modes in the native model and model with AP3 and CETCH integrated before and after deletion of the PLGG1 transporter. *: PLGG1 RNAi.

*Native model*	
Mode	Reactions
N-EM1	(1 LIGHT5_ATPase)
N-EM2	(1 PR4_CAT1)
N-EM3	(1 CBB10_RPIA) (3 CBB11_PRK) (2 CBB12_RPE) (2 CBB13_TPI) (3 CBB1_RuBisCO1) (1 CBB4_FBPaldo) (1 CBB5_FBPase) (1 CBB6_TKTL1) (1 CBB7_FBPaldo2) (1 CBB8_SBPase) (1 CBB9_TKTL2)
N-EM4	(2 CBB2_PGK) (2 CBB3_GAPDH) (1 LIGHT1_PSII) (2 LIGHT2_CYTB6F) (4 LIGHT3_PSI) (2 LIGHT4_FNR)
N-EM5	(2 CBB10_RPIA) (6 CBB11_PRK) (4 CBB12_RPE) (4 CBB13_TPI) (2 CBB4_FBPaldo) (2 CBB5_FBPase) (2 CBB6_TKTL1) (2 CBB7_FBPaldo2) (2 CBB8_SBPase) (2 CBB9_TKTL2) (3 PR10_GLYK) (6 PR1_RuBisCO2) (6 PR2_PGP) (6 PR3_GO) (3 PR5_GGT) (3 PR6_SHMT) (3 PR7_GDC) (3 PR8_AGXT) (3 PR9_HPR) (6 TR^1^_PLGG1) (6 TR^2^_TR6) (3 TR3_TR10) (3 TR^4^_PLGG1)
***Native model. (PLGG1 RNAi)***	
Mode	Reactions
N-EM1*	(1 LIGHT5_ATPase)
N-EM2*	(1 PR4_CAT1)
N-EM3*	(1 CBB10_RPIA) (3 CBB11_PRK) (2 CBB12_RPE) (2 CBB13_TPI) (3 CBB1_RuBisCO1) (1 CBB4_FBPaldo) (1 CBB5_FBPase) (1 CBB6_TKTL1) (1 CBB7_FBPaldo2) (1 CBB8_SBPase) (1 CBB9_TKTL2)
N-EM4*	(2 CBB2_PGK) (2 CBB3_GAPDH) (1 LIGHT1_PSII) (2 LIGHT2_CYTB6F) (4 LIGHT3_PSI) (2 LIGHT4_FNR)
N-EM5*	(1 CBB10_RPIA) (3 CBB11_PRK) (2 CBB12_RPE) (2 CBB13_TPI) (1 CBB4_FBPaldo) (1 CBB5_FBPase) (1 CBB6_TKTL1) (1 CBB7_FBPaldo2) (1 CBB8_SBPase) (1 CBB9_TKTL2) (3 PR1_RuBisCO2) (3 PR2_PGP)
***AP3 from South et al. and the CETCH cycle from Schwander et al.***	
Mode	Reactions
J-EM1	(1 LIGHT5_ATPase)
J-EM2	(1 CBB2_PGK) (1 CBB3_GAPDH)
J-EM3	(1 PR4_CAT1)
J-EM4	(1 CBB10_RPIA) (3 CBB11_PRK) (2 CBB12_RPE) (2 CBB13_TPI) (3 CBB1_RuBisCO1) (1 CBB4_FBPaldo) (1 CBB5_FBPase) (1 CBB6_TKTL1) (1 CBB7_FBPaldo2) (1 CBB8_SBPase) (1 CBB9_TKTL2)
J-EM5	(3 AP3_CrGDH) (3 AP3_MS) (1 CBB10_RPIA) (3 CBB11_PRK) (2 CBB12_RPE) (2 CBB13_TPI) (1 CBB4_FBPaldo) (1 CBB5_FBPase) (1 CBB6_TKTL1) (1 CBB7_FBPaldo2) (1 CBB8_SBPase) (1 CBB9_TKTL2) (3 PR1_RuBisCO2) (3 PR2_PGP)
J-EM6	(1 LIGHT1_PSII) (2 LIGHT2_CYTB6F) (4 LIGHT3_PSI) (2 LIGHT4_FNR)
J-EM7	(2 CBB10_RPIA) (6 CBB11_PRK) (4 CBB12_RPE) (4 CBB13_TPI) (2 CBB4_FBPaldo) (2 CBB5_FBPase) (2 CBB6_TKTL1) (2 CBB7_FBPaldo2) (2 CBB8_SBPase) (2 CBB9_TKTL2) (3 PR10_GLYK) (6 PR1_RuBisCO2) (6 PR2_PGP) (6 PR3_GO) (3 PR5_GGT) (3 PR6_SHMT) (3 PR7_GDC) (3 PR8_AGXT) (3 PR9_HPR) (6 TR^1^_PLGG1) (6 TR^2^_TR6) (3 TR3_TR10) (3 TR^4^_PLGG1)
J-EM8	(1 AP3_MS) (1 CETCH10_MCO) (1 CETCH11_PCO) (1 CETCH12_SCR) (1 CETCH13_SSR) (1 CETCH1_CCR) (1 CETCH2_CCR2) (1 CETCH3_EPI-ECM) (1 CETCH4_EPI-MCM) (1 CETCH5_HBD) (1 CETCH6_HBS) (1 CETCH7_KAT) (1 CETCH8_MCH) (1 CETCH9_MCL)
***AP3 from South et al. and the CETCH cycle from Schwander et al. (PLGG1 RNAi)***	
Mode	Reactions
J-EM1*	(1 LIGHT5_ATPase)
J-EM2*	(1 CBB2_PGK) (1 CBB3_GAPDH)
J-EM3*	(1 PR4_CAT1)
J-EM4*	(1 CBB10_RPIA) (3 CBB11_PRK) (2 CBB12_RPE) (2 CBB13_TPI) (3 CBB1_RuBisCO1) (1 CBB4_FBPaldo) (1 CBB5_FBPase) (1 CBB6_TKTL1) (1 CBB7_FBPaldo2) (1 CBB8_SBPase) (1 CBB9_TKTL2)
J-EM5*	(3 AP3_CrGDH) (3 AP3_MS) (1 CBB10_RPIA) (3 CBB11_PRK) (2 CBB12_RPE) (2 CBB13_TPI) (1 CBB4_FBPaldo) (1 CBB5_FBPase) (1 CBB6_TKTL1) (1 CBB7_FBPaldo2) (1 CBB8_SBPase) (1 CBB9_TKTL2) (3 PR1_RuBisCO2) (3 PR2_PGP)
J-EM6*	(1 LIGHT1_PSII) (2 LIGHT2_CYTB6F) (4 LIGHT3_PSI) (2 LIGHT4_FNR)
J-EM7*	(1 AP3_MS) (1 CETCH10_MCO) (1 CETCH11_PCO) (1 CETCH12_SCR) (1 CETCH13_SSR) (1 CETCH1_CCR) (1 CETCH2_CCR2) (1 CETCH3_EPI-ECM) (1 CETCH4_EPI-MCM) (1 CETCH5_HBD) (1 CETCH6_HBS) (1 CETCH7_KAT) (1 CETCH8_MCH) (1 CETCH9_MCL)

We based our subsequent analysis on the native model in order to envisage the realization of synthetically designed new carbon fixation mechanisms and photorespiratory bypasses in plants. We analyzed how these pathways changed the elementary modes and investigated their biological compatibility with the native model.

We first analyzed the alternative pathway 3 (AP3) from South et al. (2019), (Supplementary Figure S2), which is among the best synthetically designed but *in vivo* realized pathways, and has already been tested in tobacco under field conditions. AP3 converts glycolate to malate that can then be converted to pyruvate and acetyl-CoA in the chloroplast. The calculation of AP3 elementary modes (A-EMs) revealed a new elementary mode (A-EM5). A-EM5 starts with the oxygenase activity of Rubisco to produce 3-PGA that is then used in the CBB cycle (Supplementary Table S4). The elementary mode A-EM5 is highly influenced by the alternative pathway which leads to malate production from glycolate in the chloroplast. However, Rubisco-mediated oxygenation also leads to the photorespiratory pathway represented in A-EM6. (Supplementary Table S4).

Next, we added the CETCH cycle, an *in vitro* carbon fixation cycle developed by Schwander et al. (2016) (Supplementary Figure S3), to the native model. Incorporation of this synthetic cycle also created a new elementary mode (C-EM7), which is fixing CO_2_ to generate glyoxylate in 14 reaction steps (Supplementary Table S4). It splits N-EM4 of the native model into two separate elementary modes (C-EM2 and C-EM5). N-EM4 in the native model represents the light-dependent reactions and their connection to the CBB cycle through the production of NADPH. In the CETCH-integrated model however, the connection is lost since NADPH and NADH are now defined as external (pool) metabolites because of their iterative usage. Bifurcation of N-EM4 into C-EM2 and C-EM5 was therefore expected because the CETCH cycle has its own NADPH regeneration system (formate dehydrogenase, not shown in the model). There is a high possibility that both CETCH and CBB cycles will cross talk over the production of overlapping metabolites such as NADPH. We consider NADPH as an external metabolite; therefore, CETCH and CBB cycles behave independently from the light-dependent reactions. It is noteworthy to mention that NADPH has more than three connections, and hence, we designated it as an external metabolite. However, if NADPH is set internal, the cross talk among CBB, light-dependent reactions, and CETCH can be directly calculated by identifying the balancing elementary modes.

**FIGURE 2:**
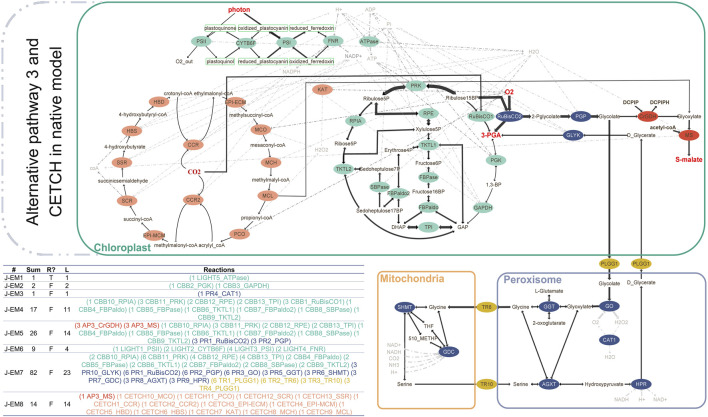
AP3 (red) and CETCH cycle (orange) integrated in the native model of photosynthesis (green) and photorespiration (blue) with transporters between organelles (gold). The elementary modes are calculated according to reversibility and metabolite classification (internal/external). Reversible reactions are shown with double-sided arrows. Sum: flux sum, R: reversibility, L: length.

Last, we integrated both CETCH cycle and AP3 in the native model (Figure 2). Combining the two synthetic pathways required selecting the suitable malate synthase (MS). In this model, we used malate synthase from AP3 (*C. maxima*), which is phylogenetically closer than *E. coli* MAS. Elementary mode analysis resulted in eight joint EMs (J-EMs). Interestingly, J-EM8 consisting of 13 reactions of the CETCH cycle and 1 reaction from AP3 represents the connection between the two synthetic pathways. We also obtained the same EM (A-EM5) with AP3 connecting the CBB cycle and the first part of the chloroplast pathway of photorespiration to AP3 (Table 1). Taken together, the introduction of synthetic cycles to the native background generates additional modes in plant CO_2_-sequestration networks.

### Modeling the Effect of the Inhibited Glycolate Flux to Peroxisome From the Chloroplast Under Native, AP3 or CETCH Cycles as Well as Combinatorial States

The occurrence of futile cycles in plants such as photorespiratory pathways initiated by the oxygenase activity of the Rubisco wastes already fixed CO_2_ in the system. South et al. (2019) applied the RNA interference approach to decrease the flux of glycolate into the native photorespiration pathway by blocking the PLGG1 transporter (plastidic glycolate/glycerate transporter 1). This results in the blockage of glycolate transportation from the chloroplast to peroxisome and the transportation of glycerate in the opposite direction. More intriguingly, the combination of AP3 with PLGG1 RNAi showed increased biomass and light-use efficiency in the field-grown tobacco transgenic plants (South et al., 2019). Keeping the effectiveness of the glycolate blockage into the peroxisome, we also incorporated this scenario in our models. In essence, these synthetic modifications are expected to bring considerable changes in the topological behavior of the models.

For the sake of convenience, we deleted PLGG1 that completely diminished the flux of metabolites into peroxisome by the process of photorespiration. All elementary modes except N-EM5 in the native model were retained upon deletion of PLGG1. It is noteworthy to mention that deletion of PLGG1 resulted in the reduction of N-EM5 to a length of 12 enzymes localized in the chloroplast. In this scenario, glycolate is still produced by the first two steps of photorespiration and no longer entering into the peroxisome (Table 1). A similar effect was also observed in the AP3-integrated native model with the flux of glycolate to peroxisome being blocked. Interestingly, A-EM6 was completely eliminated from the network in the absence of the PLGG1 transporter. As a consequence, unlike the native model, the glycolate produced in the chloroplast is then converted to malate by AP3 instead of being accumulated in the chloroplast (Supplementary Table S4).

On the other hand, the CETCH-integrated native model with the deleted PLGG1 transporter behaves similar to the native model with the blocked transporter (Supplementary Table S4). These arrangements bring modifications to the C-EM6, which now includes mainly the chloroplastic reactions with the capacity to produce glycolate (Supplementary Table S4). When PLGG1 was removed from the native model after being extended by synthetic AP3 and CETCH cycles, we observed the elimination of the longest elementary mode (J-EM7) harboring the entire photorespiratiory reactions. Moreover, the presence of AP3 in the combinatorial model leads to the production of malate from glycolate in the chloroplast (Table 1). These results substantiate the fact that the blockage of glycolate from the chloroplast to peroxisome exterminates elementary modes from the network otherwise found in the peroxisome.

### Modeling the Effect of Synthetic Cycles on the Activities of the Enzymes Involved in CO_2_ Sequestration in Plants

To assess changes in flux distributions upon the addition of synthetic pathways and/or deletion of the PLGG1 transporter, we calculated individual enzyme activities with YANAsquare considering the activity of all EMs as well as the enzyme coefficients in the EMs (Supplementary Table S5, Supplementary Table S6). The resulting enzyme activities represent the flux strength carried by each enzyme. We used this attribute to interpret the effects of modifications we performed in our models. It is noteworthy to mention that the flux carried by each enzyme is calculated based on the assumption that each EM operates at an equal activity level. With future transcriptome or even metabolite data of the anticipated synthetically designed plants, these calculations can be refined as shown previously for bacteria (Yang et al., 2019).

In light-dependent reactions, the addition of CETCH and/or AP3 increased the activity of the enzymes only when they are combined with PLGG1 deletion. Especially, the integration of the CETCH cycle with PLGG1 deletion resulted in the highest enzyme activities in this module (+75%). Likewise, the CBB cycle activities increased with the integration of AP3 and CETCH combination (6.5%) as well as the integration of AP3 alone (7.5%). The integration of the CETCH cycle alone caused a slight decrease (0.8%) in the enzyme activities of the CBB cycle with a notion that the presence of CETCH caused repression in the activities of CBB enzymes. However, highest activities for all CBB cycle enzymes were observed when addition of the CETCH cycle was combined with PLGG1 deletion (Supplementary Table S5). In summary, higher stimulation in the activities of photosynthetic reactions was achieved by almost all synthetic modifications in the absence of the functional glycolate transporter (PLGG1), which will culminate in increased CO_2_ accumulation in the chloroplast.

On the contrary, the enzyme activities of the photorespiratory pathway are repressed when PLGG1 is fully suppressed irrespective of the model tested in our analysis. The exception was the central redox buffering enzyme catalase which increased in activity. Even in the presence of PLGG1, integration of AP3 and/or CETCH decreased the activities of all photorespiration enzymes except those in the chloroplast, which increased upon the integration of the AP3 bypass. AP3 relies on the production of glycolate through the first two steps of the photorespiratory pathway (Supplementary Table S5). It is noteworthy to mention that for two enzymes of AP3, we observed an opposite behavior in enzyme activities upon the integration of the CETCH cycle. Addition of the CETCH cycle did not drastically change the activity of glycolate dehydrogenase (CrGDH) (−0.9%, −4% with and w/o PLGG1) but increased the activity of malate synthase (MS) (+32% and +28% with or w/o PLGG1) since glyoxylate production increased. The activities of CETCH cycle enzymes decreased by 20% in the presence of the AP3 bypass. However, PLGG1 deletion led to a 77 and 105% increase in CETCH enzyme activities with or without AP3, respectively (Supplementary Table S5).

Overall, modeling the effect of the topological changes on enzyme activities demonstrated a high stimulation of photosynthesis and elimination of photorespiration in peroxisome when CETCH integration is combined with PLGG1 deletion. However, effects of a 100% PLGG1 suppression should also be considered for possible toxicity that may arise from glycolate production in the chloroplast (see *Inter-pathway Compatibility, CO*_*2*_ *Harvesting Efficiency*, *and Prospects for In Planta Realization*). Thus, these *in silico* results suggest that the integration of CETCH and AP3 along with a PLGG1 deletion has stimulating effects on photosynthetic reactions and diminishing effects on photorespiratorion. Furthermore, the combination of the CETCH cycle with the AP3 bypass increased the activity of malate synthase pointing to higher malate production with implications for higher biomass formation.

### Evaluation of the Pathway Effects and Alternatives to the CETCH Cycle and AP3 Bypass

Low photosynthetic efficiency of the plants is often associated with lower carboxylase and higher oxygenase activity of the Rubisco enzyme. The higher affinity of the enzyme for oxygen leads to the energetically wasteful process of photorespiration. Many attempts have been made so far to engineer Rubisco to have a higher carboxylase activity, exchanging it with more efficient CO₂-fixing enzymes and/or full synthetic pathways, as well as implementing bypasses in photorespiration to avoid energy loss (Löwe and Kremling, 2021). Our topological analysis addresses these issues with the aim of finding optimal pathways with better efficiency and low fitness cost. We found that integration of the CETCH cycle and/or AP3 redirects the flux away from the photorespiratory reactions that occur in the peroxisome. An additional deletion of the PLGG1 transporter blocks the transportation of glycolate to peroxisome, thereby diminishing any possible metabolic path to the peroxisome (Table 1). The analyzed synthetic modulations bring changes in the topology of the CO_2_-fixation network (Figure 2) with concomitant changes in individual enzyme activities (Supplementary Table S5).

We next evaluated how robust this change is concerning the alternative pathways investigated. We integrated another recently designed alternative pathway: the GOC (glycolate oxidase–oxalate–catalase) bypass (Shen et al., 2019), which is analogous to AP3 in oxidizing glycolate into CO₂ in the chloroplast by deploying three enzymes: glycolate oxidase, oxalate oxidase, and catalase. Unlike the AP3 bypass with heterologous enzymes, the GOC pathway directly implements a bypass in rice chloroplasts using self-originating enzymes that do not produce reducing equivalents but generate reactive oxygen species (ROS) in the form of H₂O₂. It is noteworthy to mention that the GOC bypass requires 20 ATP to redirect the glycolate from photorespiration, and thus is more energy intensive than other bypass pathways (Shen et al., 2019). We calculated a requirement of 15 ATPs for AP3 according to the method from the study by Peterhansel et al. (2013), which makes AP3 more energy efficient than the GOC bypass. We also integrated the GOC pathway instead of AP3 to the native model along with the CETCH cycle (Supplementary Figure S4). We observed 9 EMs, six of which are the same as the AP3 bypass. Moreover, we observed the flux directed into the GOC pathway followed the Rubisco’s reaction with oxygen. However, the GOC pathway differed as it did not create an EM connected to the CETCH cycle since there are no connections between the two pathways except the possibility of the CETCH cycle fixing the CO₂ produced by GOC. It is still not known the CO_2_ produced by the GOC or AP3 bypass will be fixed by the CBB cycle in the native model (by the suggested CETCH cycle) or is directly released to the environment (see Supplementary text). Overall, we argue that AP3 is more suitable for co-integration with CETCH since it can deal with the glyoxlate produced in the CETCH cycle unlike the GOC cycle that does not consume glyoxylate; consequently, AP3 attains higher energy efficiency than GOC. Nevertheless, these aspects merit detailed investigation for *in vivo* realization.

Likewise, the CETCH cycle is not the only carbon-fixation alternative. It can also be replaced with very simple carbon-fixation cycles with four to six reactions (e.g., KGS-ICDH and the KGS-KGC cycles) or longer but more energetically feasible MOG (malonyl–CoA–oxaloacetate–glyoxylate) cycles. The simplest cycles found by Bar-Even et al. (2010) are not promising candidates in terms of their energy efficiency and the enzymes involved. On the other hand, although not as energetically efficient as the CETCH cycle, MOG cycles were shown to be still 2–3 times more efficient than the native CBB cycle, and their integration may be easier than the CETCH cycle due to the lower number of reactions (Naseem et al., 2020). However, MOG cycles do not fix CO₂ but HCO3- by PEP carboxylase, and they resemble more the C4 mode of carbon fixation. The output of MOG cycles is glyoxylate as in the CETCH cycle; therefore, its integration to the native model with AP3 resembles the integration of CETCH (not shown). In an eventuality where CETCH is prioritized over other CCC cycles, a thorough dynamic analysis of the models (Figure 2; Supplementary Figure S5) involving the CETCH cycle should be accomplished. The *in vitro* realization of the CETCH cycle by Schwander et al., reported enzyme-specific activity kinetics. Their analysis showed highest activities for catalase (KAT, 11,740 Umg^−1^) and mesaconyl-CoA hydratase (MCH, 1,500 Umg^−1^), while 4-hydroxybutyryl-CoA synthetase (HBS, 3.9 Umg^−1^) and methylsuccinyl-CoA oxidase (MCO. 0.1 Umg^−1^) proved to be less active. One may tempt to speculate that lower specific enzyme activities in the CETCH may limit the efficiency of the whole CETCH because our analysis (Supplementary Table S5) suggests equal flux distribution across all CETCH cycle enzymes. For future analysis, the enzyme kinetic data (Supplementary Table S5) from the study of Schwander et al. (2016) can be mapped to the CETCH cycle, and then enzyme activity estimates, network topology, and mode arrangements can be assessed.

Furthermore, the CETCH cycle also employs a catalytically more efficient (2–4 fold) CO_2_-fixing CCR enzyme (crotonoyl-coA carboxylase/reductase) than Rubisco (Schwander et al., 2016). To further demonstrate the efficiency of CETCH enzymes, we assigned two-fold activation (to mimic the higher efficiency of CCRs) to the elementary mode C-EM7 (which spans all the enzymes of the CETCH cycle) to model the effect of the CETCH cycle on flux distribution across the chloroplast, peroxisome, and mitochondrial compartments. Two-fold activation in the status of CETCH cycle enzymes leads to the activation of AP3 enzyme MS while repressing the activities of photorespiration and the CBB cycle (Supplementary Table S7). These results support the notion that CBB and CETCH cycles compete for CO_2_ within the cell with a sense of antagonism between native and synthetic cycles when the glycolate transporter is not functional. However, to fully assess this, a detailed *in vivo* analysis of the interaction between CETCH and CBB cycles should be performed. One may tempt to speculate that for boosted CO_2_ sequestration, the targeted elimination of some of the native CBB cycle enzymes with the *CRISPR-Cas* system will further enhance the capacity of the CETCH CCR due to abundance in the availability of CO_2_ as the substrate which is otherwise taken up by the native CBB enzyme in the cell.

### Inter-pathway Compatibility, CO_2_ Harvesting Efficiency, and Prospects for *In Planta* Realization

When the photorespiratory pathway is active in plants, the CBB cycle enzyme activities slightly decreased with the integration of the CETCH cycle. When the native CBB and the synthetic CETCH cycles are present in one metabolic network, we observed higher enzyme activities for the CBB cycle than the CETCH cycle, indicating the preference of CBB over CETCH by the system. This points to the notion that *in planta* realization of synthetic cycles may demand the repression of endogenous cycles in order to achieve prominent CO_2_ fixation potential of the synthetic cycles. The artificial pathways change the elementary modes in the model and hence the distribution of fluxes across the network. CBB cycle enzymes have higher estimated activity as they are present in more EMs (J-EM2,4,5,7) than CETCH cycle enzymes (J-EM8). It is because of the fact that CBB cycle enzymes have higher connectivity with other pathways in the cellular system than the artificial CETCH cycle. It is noteworthy to mention that actual kinetic data such as enzyme and metabolite concentrations or at least gene expression data (Yang et al., 2019) may help us to reveal the actual power of the CETCH cycle as without these data, currently, the equal-EM activities’ assumption overrides the actual levels of enzyme activities in the cellular system.

*In vitro* analysis of the CETCH proved that these enzymes are more energy efficient than those of the CBB cycle, (Löwe and Kremling, 2021). Our analyses are unique in studying the impact of various artificial cycles and even their combinatorial possibilities that are assessed with due pros and cons. In a scenario where CBB is eliminated from the network, the activity profile of the CETCH cycle increased by 68% (Supplementary Figure S5). Therefore, we concluded that the presence of the CBB cycle limited the use of the CETCH cycle since CBB is naturally fully integrated into the system, and its absence allowed to rescue the CETCH cycle activities. However, the replacement of CBB with CETCH may lead to major spatiotemporal changes in the plant photosynthetic system concerning metabolite balance and toxicity in the chloroplast (Supplementary text, Supplementary Figure S5). Likewise, the impairment of photorespiration in favor of CETCH implementation should have developmental consequences for the growth of the plants as the interplay between photorespiration and nitrogen as well as sulfur fixation pathways might be hampered. Hence, we recommend for *in planta* testing of such modifications using an inducible CETCH cycle, and subsequent inducible inhibition of the natural CBB cycle is highly required.

According to our enzyme activity estimations, the integration of CETCH with PLGG1 deletion showed the most stimulating effect on photosynthetic enzyme activities, while AP3 integration alone or in combination with CETCH under the deleted PLGG1 transporter showed promising effects. To assess the suitability of the artificial cycle with regard to the natural CBB cycle, we applied multiple approaches. The *in planta* realization of these cycles will lead us to produce various sets of experimental data which would further be used to rectify the output of our models. Furthermore, it would be interesting to see how system states change if we implement partial suppression of the transporter in our model to simulate the findings of South et al. (2019) with 80% repression of the transporter activity. We conducted our analysis with 100% inhibition of the glycolate transportation to the peroxisome, acknowledging the fact that complete inhibition of photorespiration might be detrimental for plants (Peterhansel et al., 2013). It is also of concern that how well the system would work in the absence of PLGG1 that would lead to the accumulation of glycolate in the chloroplast, which is toxic to the plant (Shim et al., 2019; South et al., 2019; Cui et al., 2021). In this context, 80% suppression of PLGG1 in South et al. (2019) still allows a lower amount of photorespiratory activity, while our model inhibits all photorespiration to estimate the maximum possible effects (Supplementary text). Future analysis based on 80% inhibition of the glycolate flux to peroxisome would be interesting to compare the state of modes, network topology, and enzyme activities in various model combinations (Table 1; Figures 1, 2).

## Conclusion and Future Prospects

We investigate here *in silico* both combined models and just insertion of individual pathways for improved carbon harvesting both by elementary mode analysis (EMA) and enzyme activity estimates. Our study utilizes a well-known method in metabolic modeling, EMA (Schuster et al., 2000; Yang et al., 2019), in a novel application: We used EMA to follow changes in elementary modes after the addition of AP3 and/or CETCH and deletion of a critical glycolate transporter (PLGG1) in the photorespiratory pathway. Our topological analysis provides a helpful basis for the *in planta* realization of these CO_2_-harvesting modifications, conveniently achieved in the model plant *Arabidopsis thaliana*. Likewise, these models can also be implemented in crop plants by exploiting conventional molecular biology tools as well as new genome-editing techniques. Besides crop plants, all our results are readily transferable to the highly conserved photorespiratory and photosynthetic cycles found in algae (Alami et al., 2021; Matula and Nabity, 2021), and blue-green algae (George et al., 2020). As algae and fast-growing land plants such as bamboo grass (Devi and Singh, 2021) are fast CO_2_ harvesters even without engineering, they are often aimed for potential CO_2_-sequestration applications. Future works will integrate more experimental data and then can simulate diverse scenarios more accurately with actual kinetics and better resolution.

## Data Availability

The original contributions presented in the study are included in the article/Supplementary Material; further inquiries can be directed to the corresponding authors.
