# Supplementary material for: Topological Analysis of the Carbon-Concentrating CETCH Cycle and a Photorespiratory Bypass Reveals Boosted CO2-Sequestration by Plants
Source: Front Bioeng Biotechnol. 2021 Nov 1;9:708417. doi: 10.3389/fbioe.2021.708417 (PMC8591258; doi:10.3389/fbioe.2021.708417)
Supplement: Supplementary file 1 [file DataSheet1.pdf]

Supplementary Material

We give here additional data on our pathway modelling. The native model is described in the paper and encompasses the central metabolism of photosynthesis and carbon fixation exemplified for *A. thaliana*. Modifications include integration of CETCH (crotonyl-CoA/ethylmalonyl-CoA/hydroxybutyryl-CoA) cycle, AP3 (alternative pathway 3), GOC (glycolate oxidase-oxalate-catalase) bypass and PLGG1 (plastidic glycolate/glycerate transporter 1) suppression. All ten network files in Metatool and SBML format will be fully available upon manuscript acceptance.

Table S1: Reactions in the native model.

Table S2. Metabolite states in each model.

Table S3. Net reactions of each EM in each model

Table S4: Elementary modes in models with AP3 and CETCH before and after deletion of PLGG1 transporter

Table S5: Individual enzyme activities calculated assuming each EM is fully active

Table S6: Flux distributions in native model upon deletion of glycolate transporter PLGG1.

Table S7. Flux distribution when CETCH expected efficiency is integrated.

Figure S1. Flux balance analysis in a nutshell

Figure S2. Alternative pathway 3 (AP3) from South et al. (2019) integrated to the native model.

Figure S3. CETCH cycle from (Schwander et al., 2016)) integrated to the native model.

Figure S4. CETCH cycle from Schwander et al. (2016) integrated CBB cycle and GOC pathway from Bar-Even et al. (2010) integrated to the model.

Figure S5. CETCH cycle from Schwander et al. (2016) replaced CBB cycle and AP3 integrated to the model.

Supplementary text

1 Elementary Mode Analysis (EMA) and Calculation of Flux Distributions

For the flux balance calculation, each flux mode should be balancing all internal metabolites involved (Figzre S1). For instance, one enzyme produces a product from a source metabolite and a second enzyme takes up this product as its substrate and produces a drain metabolite. Then these two enzymes form an elementary mode as they balance all internal metabolite (in this example just one) and connect the external source metabolite with the external drain metabolite. However, if one enzyme converts one external metabolite as substrate into another external metabolite as product, then the elementary mode involves no internal metabolites and only one enzyme. For catalase (CAT) in the peroxisome, the reason was that the exchange between CAT substrate is H_2O_2 produced by GO (glycolate oxidase) and CAT's product O_2 is used by GO, so the two enzymes form a closed loop (futile cycle), but if you set both substrate and product as external, being high quantity pool metabolites then you again obtain a short flux mode of just one enzyme connecting two external metabolites.

If you consider now a long, linear pathway such as the glycolysis you just get a linear chain of reactions, all internal metabolites are balanced, but the source metabolite (glucose) has always to be provided and the drain metabolite (depending on the organism pyruvate or lactate) will be external or buffered (secreted lactate, if it is yeast, or consumed in later pathways such as citric acid cycle if it is pyruvate.

Only for larger and more pathways, or more metabolites, you obtain a real network of internal metabolites and then all internal metabolites involved have to be balanced by the enzyme pathway chosen, otherwise this is no stable pathway in equilibrium. Accordingly, it is true that an elementary mode is not necessarily the "shortest" pathway, it just needs all its internal metabolites to be balanced and not further decomposable

The division of metabolites between internal and external is just according to whether they must be buffered by the enzyme reactions considered and are hence inside the network or whether they are sufficiently buffered by many reactions or occur in high quantity (so called currency metabolites, e.g. ATP). All currency metabolites and most of the metabolites we set as external are pool metabolites as they occur in 4 reactions or more. This threshold works well in practice (Cecil et al., 2015)

If the elementary modes and the pathways are calculated, they only provide an overview on all metabolic pathways accessible to the organism. To identify, how active these pathways are in a concrete situation, one can either consider data on individual enzyme activities as measured and known or should map omics data sets on the network and use then software such as YANA (Schwarz et al., 2005) or YANAsquare (Schwarz et al., 2007) to calculate the flux strengths according to these data (Cecil et al., 2015).

2 AP3 integration

AP3 is rather an alternative pathway than a bypass. It resembles the bypass pathway from Maier *et* al. (Maier et al., 2012) in a way that it does not feedback into the Calvin-Benson-Bassham (CBB) cycle but redirects flux to an alternative direction. Maier pathway completely oxidizes glycolate to CO_2 in the chloroplast and is rather controversial in its effect on photosynthetic rate and energy demand (Peterhansel et al., 2013;Xin et al., 2015). The CO_2 produced from this pathway can be either released

to the atmosphere or refixed by Rubisco and the success of this pathway largely depends on whether the benefit of increased CO_2 concentration in chloroplast will be lost by atmospheric release. A similar concern can be raised for AP3. AP3 is based on a similar idea but replaces glycolate oxidation step catalyzed by GO and CAT with glycolate dehydrogenase (GDH from *C. reinhardtii* (mentioned as CrGDH from this point on). Another difference is that AP3 uses the malate synthase (MS) from *C. maxima* whereas Maier pathway has MS from *E. coli*. Here, it is worth to mention that CETCH cycle also uses *E. coli* MS (MAS), and we did not include it in our model since combination with AP3 removes the need for the phylogenetically more distant malate synthase. GDH was already used in another bypass pathway (Kebeish et al., 2007). In this pathway, Kebeish *et* al. used *E. coli* GDH but the issue with this was the low expression of subunit F of GDH limiting growth of the transgenic plant. Furthermore, GDH having three subunits makes it more difficult to transgenically express all subunits. For this, *C. reinhardtii* GDH enzyme was identified as a single subunit enzyme by Nakamura *et* al. and AP3 uses the GDH from *C. reinhardtii* for its less complicated expression. (Peterhansel et al., 2013).

Compared to Maier pathway, GDH may be a better option than GO for conversion of glycolate to glyoxylate, because GDH uses organic co-factors as electron acceptors rather than oxygen and produces reducing power in the chloroplast (Aboelmy and Peterhansel, 2014). However, with rising temperature O_2 fixation rate of Rubisco increases as well as the energy production from light-dependent reactions. Therefore, the electron transport chain would need more oxidizing agents/electron sinks to reduce instead of more reducing power and glycolate oxidase might make more sense in these conditions as an enzyme that uses reducing power instead of producing more like glycolate dehydrogenase (Peterhansel et al., 2013). Under high-light conditions, it may be also more feasible to implement GOC pathway from Shen *et* al. (Shen et al., 2019) that does not produce reducing equivalents. In our model CrGDH is represented as using dichlorophenol indophenol (DCPIP), an artificial electron acceptor. This may raise the requirement of DCPIP addition to the model, whenever AP3 is integrated but South *et* al. (2019) also predicts that CrGDH may use the electron transport chain as an acceptor instead of an artificial one.

In our model, we decided to represent AP3 integration until the production of malate for simplicity reasons. Although our decision does not make a significant change computationally, it may raise an issue in biological context. To produce malate, acetyl-CoA is used by MS and there is no source of acetyl-CoA in our model. However, as depicted in South *et* al. (2019) and Maier pathway, malate is then converted to pyruvate that is then converted back to acetyl-CoA. Both steps release CO_2, a major potential benefit of AP3, and supplies back into the alternative pathway. These steps are catalyzed by endogenous enzymes malic enzyme (ME) and pyruvate dehydrogenase (PDH) in the chloroplast (Peterhansel et al., 2013). Although we did not include this in our model since we want to show the integration of foreign/synthetic pathway, AP3 can be well integrated into the biological system via endogenous enzymes that supply AP3 with acetyl-CoA.

3 CETCH integration

First, in absence of CBB cycle, 3-PGA produced by Rubisco and photorespiratory pathway will accumulate, while Ribulose-1,5-biphosphate will not be regenerated. Furthermore, CETCH cycle intermediates are not native to the biological system and some are toxic to the cell e.g., glyoxylate, succinate semialdehyde and propionyl-CoA (Löwe and Kremling, 2021). Glyoxylate can be utilized if AP3 is also integrated but the rest may pose problems. When compared with CBB cycle in terms of energy efficiency and activity, CETCH cycle may be superior to CBB cycle (Löwe and Kremling, 2021); however, some issues are not considered when comparing synthetic CO_2 fixation pathways to the native one. The CO_2-fixation pathways computationally analyzed by Löwe and Kremling use

vitamin B12 as a cofactor, except CBB cycle. However, plants do not have biosynthesis pathway for vitamin B12, which can make integration of CETCH cycle to a plant system not very feasible and dependent on external addition of vitamin B12 (Löwe and Kremling, 2021).

4 PLGG1 deletion

When PLGG1 is removed from the model, glycolate and glycerate in chloroplast and peroxisome are left with only one connection. This means, glycolate is produced by one reaction in chloroplast and used by one reaction in peroxisome but since we depicted them as two separate metabolites (Glycolate_H and Glycolate_P), they are now topologically external. The same case applies to glycerate, only vice versa: it's produced in peroxisome and used in chloroplast. Only when AP3 is integrated in the model, glycolate in chloroplast is still internal since it's produced by PGP and used by CrGDH.

However, we also took the biological meaning into account when determining the external/internal status of these metabolites. We can say for sure, the produced glycolate in chloroplast is external because, here we want to represent that even though PLGG1 is blocked or removed, Rubisco will keep fixing oxygen and producing glycolate in the chloroplast and in absence of PLGG1, glycolate is one of the outputs of the system. We also set the glycerate in peroxisome as external since it may be still produced although not expected in this model when there is no input for the photorespiratory reactions in peroxisome and mitochondria that would normally lead to production of glycerate. We should also mention that in our models, the status of Glycerate in peroxisome does not change the EMs since there is no EM in peroxisome in absence of PLGG1. Nevertheless, for consistency, and not to be biased, we set glycerate_P external to see if there is any other way for it to be produced although we did not expect any activity of photorespiration in this case.

We still kept the remaining metabolites as internal although they have only one connection. Since our model does not include any other reaction that may produce glycerate in chloroplast or glycolate in peroxisome, it is not logical to set them as external. In other words, our model depends on the transport of these two metabolites between chloroplast and peroxisome and in absence of PLGG1, we do not want to assume or represent that these two metabolites are buffered or that they are pool metabolites etc.

Furthermore, we compared enzyme activities of the native model with only PLGG1 deletion. Here, we obtained the highest activities in light-dependent reactions and CBB cycle while photorespiration was again diminished in the peroxisome and decreased in the chloroplast. Therefore, only deletion or inhibition of PLGG1 looked like the best solution leading to better flux distributions than in models with AP3 and/or CETCH involved. Furthermore, this would be practically easier as well compared to the requirement of integration/expression of all enzymes in CETCH and/or AP3. However, there are some shortcomings of this option. As we discussed above, inhibiting photorespiration 100% is fatal for the organism (Peterhansel et al., 2013). The second point to discuss is how well the system would work in absence of PLGG1 with no new pathway to redirect the flux starting from oxygenation of RUBP by Rubisco. The absence of a new direction will lead to the accumulation of glycolate in the chloroplast, which is toxic to the plant (South et al., 2019) (Table S2).

Therefore, in absence of the PLGG1 transporter it may be better to integrate the AP3 to further process glycolate into malate. Apart from dealing with glycolate accumulation in our PLGG1 absent model, AP3, as most of the photorespiration bypass pathways, is also good for reducing nitrogen loss caused

by photorespiration pathway. Thus, in presence of AP3, less ammonia reassimilation is required which eliminates the use of ATP and reducing power needed for reassimilation pathway (Peterhansel et al., 2010;Peterhansel et al., 2013). Technically AP3 is not a bypass but an alternative pathway since bypass pathways usually redirect the flux through a shorter pathway to obtain the same product, in photorespiration 3-PGA. AP3, on the other hand, does not produce 3-PGA but malate. This can lead to the concern that on the contrary to other photorespiratory bypass pathways (Kebeish et al., 2007;Carvalho Jde et al., 2011), AP3 may deplete CBB cycle from 3-PGA. In our model, addition of AP3 was observed to increase the CBB cycle enzymes except for Rubisco, PGK, and GAPDH.

Table S1: Reactions in the native model. Extensions of the metabolites show the localization; H:chloroplast, P:peroxisome, M:mitochondria

Module	Enzyme	Reaction
CBB cycle	RuBisCO1	CO2_H + H2O_H + Ribulose15BP_H = 2 3-PGA_H + 2 Hplus_H
CBB cycle	RPIA	Ribose5P_H = Ribulose5P_H
CBB cycle	PRK	ATP_H + Ribulose5P_H = ADP_H + Ribulose15BP_H
CBB cycle	RPE	Xylulose5P_H = Ribulose5P_H
CBB cycle	TPI	GAP_H = DHAP_H
CBB cycle	PGK	3-PGA_H + ATP_H = 1,3-BP_H + ADP_H
CBB cycle	GAPDH	1,3-BP_H + Hplus_H + NADPH_H = GAP_H + NADPplus_H + PO4_H
CBB cycle	FBPaldo	DHAP_H + GAP_H = Fructose16BP_H
CBB cycle	FBPase	Fructose16BP_H + H2O_H = Fructose6P_H + PO4_H
CBB cycle	TKTL1	Fructose6P_H + GAP_H = Erythrose4P_H + Xylulose5P_H
CBB cycle	FBPaldo2	DHAP_H + Erythrose4P_H = Sedoheptulose17BP_H
CBB cycle	SBPase	H2O_H + Sedoheptulose17BP_H = PO4_H + Sedoheptulose7P_H
CBB cycle	TKTL2	GAP_H + Sedoheptulose7P_H = Ribose5P_H + Xylulose5P_H
Light-dependent reactions	PSII	2 H2O_H + hnu_H + 2 plastoquinone_H = O2_out + 2 plastoquinol_H
Light-dependent reactions	CYTB6F	2 oxidized_plastocyanin_H + plastoquinol_H = 2 Hplus_H + plastoquinone_H + 2 reduced_plastocyanin_H
Light-dependent reactions	PSI	hnu_H + oxidized_ferredoxin_H + reduced_plastocyanin_H = oxidized_plastocyanin_H + reduced_ferredoxin_H
Light-dependent reactions	FNR	Hplus_H + NADPplus_H + 2 reduced_ferredoxin_H = NADPH_H + 2 oxidized_ferredoxin_H
Light-dependent reactions	ATPase	ADP_H + Hplus_H + PO4_H = ATP_H + H2O_H
Photorespiration	RuBisCO2	O2_H + Ribulose15BP_H = 2-Pglycolate_H + 3-PGA_H
Photorespiration	GLYK	ATP_H + D_Glycerate_H = 3-PGA_H + ADP_H + Hplus_H
Photorespiration	PGP	2-Pglycolate_H + H2O_H = Glycolate_H + PO4_H
Photorespiration	GO	Glycolate_P + O2_P = Glyoxylate_P + H2O2_P
Photorespiration	CAT1	2 H2O2_P = 2 H2O_P + O2_P
Photorespiration	GGT	Glyoxylate_P + L-Glutamate_P = 2-oxoglutarate_P + Glycine_P
Photorespiration	SHMT	510_METHF_M + Glycine_M = Serine_M + THF_M
Photorespiration	GDC	Glycine_M + NADplus_M + THF_M = 510_METHF_M + CO2_M + Hplus_M + NADH_M + NH3_M
Photorespiration	AGXT	Glyoxylate_P + Serine_P = Glycine_P + Hydroxypyruvate_P
Photorespiration	HPR	Hplus_P + Hydroxypyruvate_P + NADH_P = D_Glycerate_P + NADplus_P
Transport	PLGG1	Glycolate_H = Glycolate_P
Transport	TR6	Glycine_P = Glycine_M
Transport	TR10	Serine_M = Serine_P
Transport	PLGG1	D_Glycerate_P = D_Glycerate_H

Table S2. Metabolite states in each model. Int: internal C: connectivity F:false T: true

Name	native		AP3		CETCH		AP3 and CETCH		AP3 (PLGG1 RNAi)		CETCH (PLGG1 RNAi)		AP3 and CETCH (PLGG1 RNAi)	
	Int	C	Int	C	Int	C	Int	C	Int	C	Int	C	Int	C
2-oxoglutarate_P	F	1	F	1	F	1	F	1	F	1	F	1	F	1
CO2_H	F	1	F	1	F	3	F	3	F	1	F	3	F	3
CO2_M	F	1	F	1	F	1	F	1	F	1	F	1	F	1
H2O_P	F	1	F	1	F	1	F	1	F	1	F	1	F	1
Hplus_M	F	1	F	1	F	1	F	1	F	1	F	1	F	1
Hplus_P	F	1	F	1	F	1	F	1	F	1	F	1	F	1
L-Glutamate_P	F	1	F	1	F	1	F	1	F	1	F	1	F	1
NADH_M	F	1	F	1	F	1	F	1	F	1	F	1	F	1
NADH_P	F	1	F	1	F	1	F	1	F	1	F	1	F	1
NADplus_M	F	1	F	1	F	1	F	1	F	1	F	1	F	1
NADplus_P	F	1	F	1	F	1	F	1	F	1	F	1	F	1
NH3_M	F	1	F	1	F	1	F	1	F	1	F	1	F	1
O2_H	F	1	F	1	F	4	F	4	F	1	F	4	F	4
O2_out	F	1	F	1	F	1	F	1	F	1	F	1	F	1
1 3-BP_H	T	2	T	2	T	2	T	2	T	2	T	2	T	2
2-Pglycolate_H	T	2	T	2	T	2	T	2	T	2	T	2	T	2
510_METHF_M	T	2	T	2	T	2	T	2	T	2	T	2	T	2
D_Glycerate_H	T	2	T	2	T	2	T	2	T	1	T	1	T	1
D_Glycerate_P	T	2	T	2	T	2	T	2	F	1	F	1	F	1
Erythrose4P_H	T	2	T	2	T	2	T	2	T	2	T	2	T	2
Fructose16BP_H	T	2	T	2	T	2	T	2	T	2	T	2	T	2
Fructose6P_H	T	2	T	2	T	2	T	2	T	2	T	2	T	2
Glycolate_H	T	2	T	3	T	2	T	3	T	2	F	1	T	2
Glycolate_P	T	2	T	2	T	2	T	2	T	1	T	1	T	1
H2O2_P	F	2	F	2	F	2	F	2	F	2	F	2	F	2
hnu_H	F	2	F	2	F	2	F	2	F	2	F	2	F	2
Hydroxypyruvate_P	T	2	T	2	T	2	T	2	T	2	T	2	T	2
NADPH_H	T	2	T	2	F	6	F	6	T	2	F	6	F	6
NADPplus_H	T	2	T	2	F	6	F	6	T	2	F	6	F	6
O2_P	F	2	F	2	F	2	F	2	F	2	F	2	F	2
oxidized_ferredoxin_H	T	2	T	2	T	2	T	2	T	2	T	2	T	2
oxidized_plastocyanin_H	T	2	T	2	T	2	T	2	T	2	T	2	T	2
plastoquinol_H	T	2	T	2	T	2	T	2	T	2	T	2	T	2

**plastoquinone_H**	T	2	T	2	T	2	T	2	T	2	T	2	T	2
**reduced_ferredoxin_H**	T	2	T	2	T	2	T	2	T	2	T	2	T	2
**reduced_plastocyanin_H**	T	2	T	2	T	2	T	2	T	2	T	2	T	2
**Ribose5P_H**	T	2	T	2	T	2	T	2	T	2	T	2	T	2
**Sedoheptulose17BP_H**	T	2	T	2	T	2	T	2	T	2	T	2	T	2
**Sedoheptulose7P_H**	T	2	T	2	T	2	T	2	T	2	T	2	T	2
**Serine_M**	T	2	T	2	T	2	T	2	T	2	T	2	T	2
**Serine_P**	T	2	T	2	T	2	T	2	T	2	T	2	T	2
**THF_M**	T	2	T	2	T	2	T	2	T	2	T	2	T	2
**DHAP_H**	T	3	T	3	T	3	T	3	T	3	T	3	T	3
**Glycine_M**	T	3	T	3	T	3	T	3	T	3	T	3	T	3
**Glycine_P**	T	3	T	3	T	3	T	3	T	3	T	3	T	3
**Glyoxylate_P**	T	3	T	3	T	3	T	3	T	3	T	3	T	3
**Ribulose15BP_H**	T	3	T	3	T	3	T	3	T	3	T	3	T	3
**Ribulose5P_H**	T	3	T	3	T	3	T	3	T	3	T	3	T	3
**Xylulose5P_H**	T	3	T	3	T	3	T	3	T	3	T	3	T	3
**3-PGA_H**	F	4	F	4	F	4	F	4	F	4	F	4	F	4
**ADP_H**	F	4	F	4	F	5	F	5	F	4	F	5	F	5
**ATP_H**	F	4	F	4	F	5	F	5	F	4	F	5	F	5
**GAP_H**	F	5	F	5	F	5	F	5	F	5	F	5	F	5
**PO4_H**	F	5	F	5	F	6	F	6	F	5	F	6	F	6
**H2O_H**	F	6	F	7	F	10	F	10	F	7	F	10	F	10
**Hplus_H**	F	6	F	6	F	6	F	6	F	6	F	6	F	6
**4-hydroxybutyrate_H**	--	--	--	--	T	2	T	2	--	--	T	2	T	2
**4-hydroxybutyryl-coA_H**	--	--	--	--	T	2	T	2	--	--	T	2	T	2
**acetyl_coA_H**	--	--	--	--	F	1	F	1	--	--	F	1	F	1
**acrylyl_coA_H**	--	--	--	--	T	2	T	2	--	--	T	2	T	2
**coA_H**	--	--	F	1	F	3	F	3	F	1	F	3	F	3
**crotonyl-coA_H**	--	--	--	--	T	2	T	2	--	--	T	2	T	2
**DCPIP_H**	--	--	F	1	--	--	F	1	F	1	--	--	F	1
**DCPIPH_H**	--	--	F	1	--	--	F	1	F	1	--	--	F	1
**ethylmalonyl-coA_H**	--	--	--	--	T	2	T	2	--	--	T	2	T	2
**Glyoxylate_H**	--	--	T	2	T	2	T	3	T	2	T	2	T	3
**H2O2_H**	--	--	--	--	T	3	T	3	--	--	T	3	T	3
**mesaconyl-coA_H**	--	--	--	--	T	2	T	2	--	--	T	2	T	2
**methylmalonyl-coA_H**	--	--	--	--	T	2	T	2	--	--	T	2	T	2
**methylmalyl-coA_H**	--	--	--	--	T	2	T	2	--	--	T	2	T	2
**methylsuccinyl-coA_H**	--	--	--	--	T	2	T	2	--	--	T	2	T	2

**propionyl-coA_H**	--	--	--	--	T	2	T	2	--	--	T	2	T	2
**S_malate_H**	--	--	--	--	F	1	F	1	--	--	F	1	F	1
**succinicsemialdehyde_H**	--	--	--	--	T	2	T	2	--	--	T	2	T	2
**succinyl-coA_H**	--	--	--	--	T	2	T	2	--	--	T	2	T	2

Table S3. Net reactions of each EM in each model. *:PLGG1 RNAi

Native model	
Mode	Net Reaction
N-EM1	ADP_H + Hplus_H + PO4_H = ATP_H + H2O_H
N-EM2	2 H2O2_P = 2 H2O_P + O2_P
N-EM3	3 ATP_H + 3 CO2_H + 5 GAP_H + 5 H2O_H = 6 3-PGA_H + 3 ADP_H + 6 Hplus_H + 2 PO4_H
N-EM4	2 3-PGA_H + 2 ATP_H + 2 H2O_H + 5 hnu_H = 2 ADP_H + 2 GAP_H + O2_out + 2 PO4_H
N-EM5	9 ATP_H + 10 GAP_H + 10 H2O_H + 3 Hplus_P + 3 L-Glutamate_P + 3 NADH_P + 3 NADplus_M + 6 O2_H + 6 O2_P = 3 2-oxoglutarate_P + 9 3-PGA_H + 9 ADP_H + 3 CO2_M + 6 H2O2_P + 3 Hplus_H + 3 Hplus_M + 3 NADH_M + 3 NADplus_P + 3 NH3_M + 10 PO4_H
Native model. (PLGG1 RNAi)	
Mode	Net Reaction
N-EM1*	ADP_H + Hplus_H + PO4_H = ATP_H + H2O_H
N-EM2*	2 H2O2_P = 2 H2O_P + O2_P
N-EM3*	3 ATP_H + 3 CO2_H + 5 GAP_H + 5 H2O_H = 6 3-PGA_H + 3 ADP_H + 6 Hplus_H + 2 PO4_H
N-EM4*	2 3-PGA_H + 2 ATP_H + 2 H2O_H + 5 hnu_H = 2 ADP_H + 2 GAP_H + O2_out + 2 PO4_H
N-EM5*	3 ATP_H + 5 GAP_H + 5 H2O_H + 3 O2_H = 3 3-PGA_H + 3 ADP_H + 3 Glycolate_H + 5 PO4_H
AP3 from South et al. and CETCH cycle from Schwander et al.	
Mode	Net Reaction
J-EM1	ADP_H + Hplus_H + PO4_H = ATP_H + H2O_H
J-EM2	3-PGA_H + ATP_H + Hplus_H + NADPH_H = ADP_H + GAP_H + NADPplus_H + PO4_H
J-EM3	2 H2O2_P = 2 H2O_P + O2_P
J-EM4	3 ATP_H + 3 CO2_H + 5 GAP_H + 5 H2O_H = 6 3-PGA_H + 3 ADP_H + 6 Hplus_H + 2 PO4_H
J-EM5	3 ATP_H + 3 DCPIP_H + 5 GAP_H + 8 H2O_H + 3 O2_H + 3 acetyl_coA_H = 3 3-PGA_H + 3 ADP_H + 3 DCPIPH_H + 5 PO4_H + 3 S_malate_H + 3 coA_H
J-EM6	2 H2O_H + 2 NADPplus_H + 5 hnu_H = 2 Hplus_H + 2 NADPH_H + O2_out
J-EM7	9 ATP_H + 10 GAP_H + 10 H2O_H + 3 Hplus_P + 3 L-Glutamate_P + 3 NADH_P + 3 NADplus_M + 6 O2_H + 6 O2_P = 3 2-oxoglutarate_P + 9 3-PGA_H + 9 ADP_H + 3 CO2_M + 6 H2O2_P + 3 Hplus_H + 3 Hplus_M + 3 NADH_M + 3 NADplus_P + 3 NH3_M + 10 PO4_H
J-EM8	ATP_H + 2 CO2_H + 4 NADPH_H + O2_H + acetyl_coA_H = ADP_H + H2O_H + 4 NADPplus_H + PO4_H + S_malate_H + coA_H
AP3 from South et al. and CETCH cycle from Schwander et al. (PLGG1 RNAi)	
Mode	Net Reaction
J-EM1*	ADP_H + Hplus_H + PO4_H = ATP_H + H2O_H
J-EM2*	3-PGA_H + ATP_H + Hplus_H + NADPH_H = ADP_H + GAP_H + NADPplus_H + PO4_H
J-EM3*	2 H2O2_P = 2 H2O_P + O2_P
J-EM4*	3 ATP_H + 3 CO2_H + 5 GAP_H + 5 H2O_H = 6 3-PGA_H + 3 ADP_H + 6 Hplus_H + 2 PO4_H
J-EM5*	3 ATP_H + 3 DCPIP_H + 5 GAP_H + 8 H2O_H + 3 O2_H + 3 acetyl_coA_H = 3 3-PGA_H + 3 ADP_H + 3 DCPIPH_H + 5 PO4_H + 3 S_malate_H + 3 coA_H
J-EM6*	2 H2O_H + 2 NADPplus_H + 5 hnu_H = 2 Hplus_H + 2 NADPH_H + O2_out

J-EM7*	ATP_H + 2 CO2_H + 4 NADPH_H + O2_H + acetyl_coA_H = ADP_H + H2O_H + 4 NADPplus_H + PO4_H + S_malate_H + coA_H
Alternative pathway 3 from South et al.	
Mode	Net Reaction
A-EM1	ADP_H + Hplus_H + PO4_H = ATP_H + H2O_H
A-EM2	2 H2O2_P = 2 H2O_P + O2_P
A-EM3	3 ATP_H + 3 CO2_H + 5 GAP_H + 5 H2O_H = 6 3-PGA_H + 3 ADP_H + 6 Hplus_H + 2 PO4_H
A-EM4	2 3-PGA_H + 2 ATP_H + 2 H2O_H + 5 hnu_H = 2 ADP_H + 2 GAP_H + O2_out + 2 PO4_H
A-EM5	3 ATP_H + 3 DCPIP_H + 5 GAP_H + 8 H2O_H + 3 O2_H + 3 acetyl-coA_H = 3 3-PGA_H + 3 ADP_H + 3 DCPIPH_H + 5 PO4_H + 3 S-malate_H + 3 coA_H
A-EM6	9 ATP_H + 10 GAP_H + 10 H2O_H + 3 Hplus_P + 3 L-Glutamate_P + 3 NADH_P + 3 NADplus_M + 6 O2_H + 6 O2_P = 3 2-oxoglutarate_P + 9 3-PGA_H + 9 ADP_H + 3 CO2_M + 6 H2O2_P + 3 Hplus_H + 3 Hplus_M + 3 NADH_M + 3 NADplus_P + 3 NH3_M + 10 PO4_H
Alternative pathway 3 from South et al. (PLGG1 RNAi)	
Mode	Net Reaction
A-EM1*	ADP_H + Hplus_H + PO4_H = ATP_H + H2O_H
A-EM2*	2 H2O2_P = 2 H2O_P + O2_P
A-EM3*	3 ATP_H + 3 CO2_H + 5 GAP_H + 5 H2O_H = 6 3-PGA_H + 3 ADP_H + 6 Hplus_H + 2 PO4_H
A-EM4*	2 3-PGA_H + 2 ATP_H + 2 H2O_H + 5 hnu_H = 2 ADP_H + 2 GAP_H + O2_out + 2 PO4_H
A-EM5**	3 ATP_H + 3 DCPIP_H + 5 GAP_H + 8 H2O_H + 3 O2_H + 3 acetyl-coA_H = 3 3-PGA_H + 3 ADP_H + 3 DCPIPH_H + 5 PO4_H + 3 S-malate_H + 3 coA_H
CETCH cycle from Schwander et al.	
Mode	Net Reaction
C-EM1	ADP_H + Hplus_H + PO4_H = ATP_H + H2O_H
C-EM2	3-PGA_H + ATP_H + Hplus_H + NADPH_H = ADP_H + GAP_H + NADPplus_H + PO4_H
C-EM3	2 H2O2_P = 2 H2O_P + O2_P
C-EM4	3 ATP_H + 3 CO2_H + 5 GAP_H + 5 H2O_H = 6 3-PGA_H + 3 ADP_H + 6 Hplus_H + 2 PO4_H
C-EM5	2 H2O_H + 2 NADPplus_H + 5 hnu_H = 2 Hplus_H + 2 NADPH_H + O2_out
C-EM6	9 ATP_H + 10 GAP_H + 10 H2O_H + 3 Hplus_P + 3 L-Glutamate_P + 3 NADH_P + 3 NADplus_M + 6 O2_H + 6 O2_P = 3 2-oxoglutarate_P + 9 3-PGA_H + 9 ADP_H + 3 CO2_M + 6 H2O2_P + 3 Hplus_H + 3 Hplus_M + 3 NADH_M + 3 NADplus_P + 3 NH3_M + 10 PO4_H
C-EM7	ATP_H + 2 CO2_H + 4 NADPH_H + O2_H + acetyl_coA_H = ADP_H + H2O_H + 4 NADPplus_H + PO4_H + S_malate_H + coA_H
CETCH cycle from Schwander et al. (PLGG1 RNAi)	
Mode	Net Reaction
C-EM1*	ADP_H + Hplus_H + PO4_H = ATP_H + H2O_H
C-EM2*	3-PGA_H + ATP_H + Hplus_H + NADPH_H = ADP_H + GAP_H + NADPplus_H + PO4_H
C-EM3*	2 H2O2_P = 2 H2O_P + O2_P
C-EM4*	3 ATP_H + 3 CO2_H + 5 GAP_H + 5 H2O_H = 6 3-PGA_H + 3 ADP_H + 6 Hplus_H + 2 PO4_H
C-EM5*	3 ATP_H + 5 GAP_H + 5 H2O_H + 3 O2_H = 3 3-PGA_H + 3 ADP_H + 3 Glycolate_H + 5 PO4_H
C-EM6*	2 H2O_H + 2 NADPplus_H + 5 hnu_H = 2 Hplus_H + 2 NADPH_H + O2_out
C-EM7*	ATP_H + 2 CO2_H + 4 NADPH_H + O2_H + acetyl_coA_H = ADP_H + H2O_H + 4 NADPplus_H + PO4_H + S_malate_H + coA_H

Table S4: Elementary modes in models with AP3 and CETCH before and after deletion of PLGG1 transporter (A-EM: AP3 elementary mode, A-EM*: AP3-PLGG1 RNAi elementary mode, C-EM: CETCH elementary mode, C-EM*: CETCH-PLGG1 RNAi elementary mode)

Alternative pathway 3 from South et al.	
Mode	Reactions
A-EM1	(1 LIGHT5_ATPase)
A-EM2	(1 PR4_CAT1)
A-EM3	(1 CBB10_RPIA) (3 CBB11_PRK) (2 CBB12_RPE) (2 CBB13_TPI) (3 CBB1_RuBisCO1) (1 CBB4_FBPaldo) (1 CBB5_FBPase) (1 CBB6_TKTL1) (1 CBB7_FBPaldo2) (1 CBB8_SBPase) (1 CBB9_TKTL2)
A-EM4	(2 CBB2_PGK) (2 CBB3_GAPDH) (1 LIGHT1_PSII) (2 LIGHT2_CYTB6F) (4 LIGHT3_PSI) (2 LIGHT4_FNR)
A-EM5	(3 AP3_CrGDH) (3 AP3_MS) (1 CBB10_RPIA) (3 CBB11_PRK) (2 CBB12_RPE) (2 CBB13_TPI) (1 CBB4_FBPaldo) (1 CBB5_FBPase) (1 CBB6_TKTL1) (1 CBB7_FBPaldo2) (1 CBB8_SBPase) (1 CBB9_TKTL2) (3 PR1_RuBisCO2) (3 PR2_PGP)
A-EM6	(2 CBB10_RPIA) (6 CBB11_PRK) (4 CBB12_RPE) (4 CBB13_TPI) (2 CBB4_FBPaldo) (2 CBB5_FBPase) (2 CBB6_TKTL1) (2 CBB7_FBPaldo2) (2 CBB8_SBPase) (2 CBB9_TKTL2) (3 PR10_GLYK) (6 PR1_RuBisCO2) (6 PR2_PGP) (6 PR3_GO) (3 PR5_GGT) (3 PR6_SHMT) (3 PR7_GDC) (3 PR8_AGXT) (3 PR9_HPR) (6 TR1_PLGG1) (6 TR2_TR6) (3 TR3_TR10) (3 TR4_PLGG1)
Alternative pathway 3 from South et al. (PLGG1 RNAi)	
Mode	Reactions
A-EM1*	(1 LIGHT5_ATPase)
A-EM2*	(1 PR4_CAT1)
A-EM3*	(1 CBB10_RPIA) (3 CBB11_PRK) (2 CBB12_RPE) (2 CBB13_TPI) (3 CBB1_RuBisCO1) (1 CBB4_FBPaldo) (1 CBB5_FBPase) (1 CBB6_TKTL1) (1 CBB7_FBPaldo2) (1 CBB8_SBPase) (1 CBB9_TKTL2)
A-EM4*	(2 CBB2_PGK) (2 CBB3_GAPDH) (1 LIGHT1_PSII) (2 LIGHT2_CYTB6F) (4 LIGHT3_PSI) (2 LIGHT4_FNR)
A-EM5**	(3 AP3_CrGDH) (3 AP3_MS) (1 CBB10_RPIA) (3 CBB11_PRK) (2 CBB12_RPE) (2 CBB13_TPI) (1 CBB4_FBPaldo) (1 CBB5_FBPase) (1 CBB6_TKTL1) (1 CBB7_FBPaldo2) (1 CBB8_SBPase) (1 CBB9_TKTL2) (3 PR1_RuBisCO2) (3 PR2_PGP)
CETCH cycle from Schwander et al.	
Mode	Reactions
C-EM1	(1 LIGHT5_ATPase)
C-EM2	(1 CBB2_PGK) (1 CBB3_GAPDH)
C-EM3	(1 PR4_CAT1)
C-EM4	(1 CBB10_RPIA) (3 CBB11_PRK) (2 CBB12_RPE) (2 CBB13_TPI) (3 CBB1_RuBisCO1) (1 CBB4_FBPaldo) (1 CBB5_FBPase) (1 CBB6_TKTL1) (1 CBB7_FBPaldo2) (1 CBB8_SBPase) (1 CBB9_TKTL2)
C-EM5	(1 LIGHT1_PSII) (2 LIGHT2_CYTB6F) (4 LIGHT3_PSI) (2 LIGHT4_FNR)

C-EM6	(2 CBB10_RPIA) (6 CBB11_PRK) (4 CBB12_RPE) (4 CBB13_TPI) (2 CBB4_FBPaldo) (2 CBB5_FBPase) (2 CBB6_TKTL1) (2 CBB7_FBPaldo2) (2 CBB8_SBPase) (2 CBB9_TKTL2) (3 PR10_GLYK) (6 PR1_RuBisCO2) (6 PR2_PGP) (6 PR3_GO) (3 PR5_GGT) (3 PR6_SHMT) (3 PR7_GDC) (3 PR8_AGXT) (3 PR9_HPR) (6 TR1_PLGG1) (6 TR2_TR6) (3 TR3_TR10) (3 TR4_PLGG1)
C-EM7	(1 CETCH10_MCO) (1 CETCH11_PCO) (1 CETCH12_SCR) (1 CETCH13_SSR) (CETCH14_MAS) (1 CETCH1_CCR) (1 CETCH2_CCR2) (1 CETCH3_EPI-ECM) (1 CETCH4_EPI-MCM) (1 CETCH5_HBD) (1 CETCH6_HBS) (1 CETCH7_KAT) (1 CETCH8_MCH) (1 CETCH9_MCL)
CETCH cycle from Schwander et al. (PLGG1 RNAi)	
Mode	Reactions
C-EM1*	(1 LIGHT5_ATPase)
C-EM2*	(1 CBB2_PGK) (1 CBB3_GAPDH)
C-EM3*	(1 PR4_CAT1)
C-EM4*	(1 CBB10_RPIA) (3 CBB11_PRK) (2 CBB12_RPE) (2 CBB13_TPI) (3 CBB1_RuBisCO1) (1 CBB4_FBPaldo) (1 CBB5_FBPase) (1 CBB6_TKTL1) (1 CBB7_FBPaldo2) (1 CBB8_SBPase) (1 CBB9_TKTL2)
C-EM5*	(1 LIGHT1_PSII) (2 LIGHT2_CYTB6F) (4 LIGHT3_PSI) (2 LIGHT4_FNR)
C-EM6*	(1 CBB10_RPIA) (3 CBB11_PRK) (2 CBB12_RPE) (2 CBB13_TPI) (1 CBB4_FBPaldo) (1 CBB5_FBPase) (1 CBB6_TKTL1) (1 CBB7_FBPaldo2) (1 CBB8_SBPase) (1 CBB9_TKTL2) (3 PR1_RuBisCO2) (3 PR2_PGP)
C-EM7*	(1 CETCH10_MCO) (1 CETCH11_PCO) (1 CETCH12_SCR) (1 CETCH13_SSR) (CETCH14_MAS) (1 CETCH1_CCR) (1 CETCH2_CCR2) (1 CETCH3_EPI-ECM) (1 CETCH4_EPI-MCM) (1 CETCH5_HBD) (1 CETCH6_HBS) (1 CETCH7_KAT) (1 CETCH8_MCH) (1 CETCH9_MCL)

Table S5: Individual enzyme activities calculated by assuming each EM is fully active[1]

Name	Full name	Native	AP3	CETCH	AP3 and CETCH	AP3 (PLGG 1 RNAi)	CETCH (PLGG 1 RNAi)	AP3 & CETCH (PLGG 1 RNAi)
Light-dependent reactions								
PSII	Photosystem II	0.0442	0.0356	0.0439	0.0353	0.0754	0.0776	0.0725
CYTB6F	Cytochrome b6f complex	0.0884	0.0712	0.0877	0.0706	0.1508	0.1552	0.1451
PSI	Photosystem I	0.1768	0.1425	0.1754	0.1412	0.3015	0.3105	0.2902
FNR	ferredoxin---NADP+ reductase	0.0884	0.0712	0.0877	0.0706	0.1508	0.1552	0.1451
ATPase	ATP synthase	0.0442	0.0356	0.0439	0.0353	0.0754	0.0776	0.0725
Calvin-Benson-Bassham Cycle								
RuBisCO1	Rubisco	0.1326	0.1069	0.1316	0.1059	0.2261	0.2328	0.2176
PGK	phosphoglycerate kinase	0.0884	0.0712	0.0439	0.0353	0.1508	0.0776	0.0725
GAPDH	glyceraldehyde-3-phosphate dehydrogenase	0.0884	0.0712	0.0439	0.0353	0.1508	0.0776	0.0725
FBPaldo	fructose 1,6-bisphosphate aldolase	0.1326	0.1425	0.1316	0.1412	0.1508	0.1552	0.1451
FBPase	fructose-1,6-bisphosphatase I	0.1326	0.1425	0.1316	0.1412	0.1508	0.1552	0.1451
TKTL1	transketolase	0.1326	0.1425	0.1316	0.1412	0.1508	0.1552	0.1451
FBPaldo2	fructose-bisphosphate aldolase, class I	0.1326	0.1425	0.1316	0.1412	0.1508	0.1552	0.1451
SBPase	sedoheptulose-bisphosphatase	0.1326	0.1425	0.1316	0.1412	0.1508	0.1552	0.1451
TKTL2	transketolase	0.1326	0.1425	0.1316	0.1412	0.1508	0.1552	0.1451
RPIA	ribose 5-phosphate isomerase	0.1326	0.1425	0.1316	0.1412	0.1508	0.1552	0.1451
PRK	phosphoribulokinase	0.3977	0.4275	0.3947	0.4237	0.4523	0.4657	0.4353
RPE	ribulose phosphate 3-epimerase	0.2652	0.285	0.2631	0.2825	0.3015	0.3105	0.2902
TPI	triose-phosphate isomerase	0.2652	0.285	0.2631	0.2825	0.3015	0.3105	0.2902
Photorespiration								
RuBisCO2	Rubisco	0.2652	0.3206	0.2631	0.3178	0.2261	0.2328	0.2176
PGP	phosphoglycolate phosphatase	0.2652	0.3206	0.2631	0.3178	0.2261	0.2328	0.2176
TR1_PLGG1	PLGG1	0.2652	0.2137	0.2631	0.2119	--	--	--
GO	glycolate oxidase	0.2652	0.2137	0.2631	0.2119	0	0	0
CAT1	catalase	0.0442	0.0356	0.0439	0.0353	0.0754	0.0776	0.0725
GGT	glycine transaminase	0.1326	0.1069	0.1316	0.1059	0	0	0
TR6	TR6	0.2652	0.2137	0.2631	0.2119	0	0	0
SHMT	Serine hydroxymethyltransferase 4	0.1326	0.1069	0.1316	0.1059	0	0	0
GDC	glycine cleavage system	0.1326	0.1069	0.1316	0.1059	0	0	0
TR10	TR10	0.1326	0.1069	0.1316	0.1059	0	0	0
AGXT	serine---glyoxylate transaminase	0.1326	0.1069	0.1316	0.1059	0	0	0
HPR	glycerate dehydrogenase	0.1326	0.1069	0.1316	0.1059	0	0	0
TR4_PLGG1	PLGG1	0.1326	0.1069	0.1316	0.1059	--	--	--
GLYK	glycerate 3-kinase	0.1326	0.1069	0.1316	0.1059	0	0	0
Alternative pathway 3 from South et al.								
CrGDH	glycolate dehydrogenase	--	0.1069	--	0.1059	0.2261	--	0.2176
MS	malate synthase	--	0.1069	--	0.1412	0.2261	--	0.2902
CETCH cycle from Schwander et al.		***Exp.****						
CCR	crotonyl-CoA carboxylase/reductase	***110***	--	0.0439	0.0353	--	0.0776	0.0725
CCR2	crotonyl-CoA carboxylase/reductase	***110***	--	0.0439	0.0353	--	0.0776	0.0725
EPI-ECM	emC/mmC epimerase&ethylmalonyl-CoA mutase	***223.5****	--	0.0439	0.0353	--	0.0776	0.0725
EPI-MCM	emC/mmC epimerase&methylmalonyl-CoA mutase	***230****	--	0.0439	0.0353	--	0.0776	0.0725
HBD	4-hydroxybutyryl-CoA dehydratase	***26***	--	0.0439	0.0353	--	0.0776	0.0725
HBS	4-hydroxybutyryl-CoA synthetase	***2***	--	0.0439	0.0353	--	0.0776	0.0725
KAT	katalase	***11740***	--	0.0439	0.0353	--	0.0776	0.0725
MCH	mesaconyl-CoA hydratase	***1500***	--	0.0439	0.0353	--	0.0776	0.0725
MCL	β-methylmalyl-CoA lyase	***5***	--	0.0439	0.0353	--	0.0776	0.0725
MCO	methylsuccinyl-CoA oxidase	***0.1***	--	0.0439	0.0353	--	0.0776	0.0725
PCO	propionyl-CoA oxidase	***12***	--	0.0439	0.0353	--	0.0776	0.0725
SCR	succinyl-CoA reductase	***29***	--	0.0439	0.0353	--	0.0776	0.0725
SSR	succinic semialdehyde reductase	***3.9***	--	0.0439	0.0353	--	0.0776	0.0725
MAS	malate synthase	***36***	--	0.0439	--	--	0.0776	--

[1]red: lowest activity of the enzyme, green: highest activity. The activities in the native model represent the basis state and addition of synthetic pathways AP3 and CETCH separately and overall changes of the individual enzyme activities. The

effect of PLGG1 RNAi is also incorporated as deletion of PLGG1 transporter and shown in the last 3 columns. *Experimental specific enzyme activities from Schwander et al. (U mg^{-1}) **average of the two enzymes shown.

Table S6. Flux distributions in native model upon deletion of glycolate transporter PLGG1.

Name	Full name	Raw flux	Normalized flux
RPIA	ribose 5-phosphate isomerase	0.8944	0.1591
PRK	phosphoribulokinase	2.6833	0.4773
RPE	ribulose phosphate 3-epimerase	1.7889	0.3182
TPI	triose-phosphate isomerase	1.7889	0.3182
RuBisCO1	Rubisco	1.3416	0.2387
PGK	phosphoglycerate kinase	0.8944	0.1591
GAPDH	glyceraldehyde-3-phosphate dehydrogenase	0.8944	0.1591
FBPaldo	fructose 1,6-bisphosphate aldolase	0.8944	0.1591
FBPase	fructose-1,6-bisphosphatase I	0.8944	0.1591
TKTL1	transketolase	0.8944	0.1591
FBPaldo2	fructose-bisphosphate aldolase	0.8944	0.1591
SBPase	sedoheptulose-bisphosphatase	0.8944	0.1591
TKTL2	transketolase	0.8944	0.1591
PSII	photosystem II	0.4472	0.0796
CYTB6F	cytochrome b6f complex	0.8944	0.1591
PSI	Photosystem I	1.7889	0.3182
FNR	ferredoxin-NADP+ reductase	0.8944	0.1591
ATPase	ATP synthase	0.4472	0.0796
GLYK	glycerate 3-kinase	0.0	0.0
RuBisCO2	Rubisco	1.3416	0.2387
PGP	phosphoglycolate phosphatase	1.3416	0.2387
GO	glycolate oxidase	0.0	0.0
CAT1	catalase	0.4472	0.0796
GGT	glycine transaminase	0.0	0.0
SHMT	serine hydroxymethyltransferase 4	0.0	0.0
GDC	glycine cleavage system	0.0	0.0
AGXT	serine-glyoxylate transaminase	0.0	0.0
HPR	glycerate dehydrogenase	0.0	0.0
TR6	TR6	0.0	0.0
TR10	TR10	0.0	0.0

Table S7. Flux distribution when CETCH expected efficiency is integrated. *: CETCH EM is given two-fold activity. We put the activities from the models when all EMs are equally active for comparison.

Name	CETCH integrated	CETCH integrated *	AP3 and CETCH integrated	AP3 and CETCH integrated *	CETCH integrated (PLGG1 RNAi)	CETCH integrated (PLGG1 RNAi) *	AP3 and CETCH integrated (PLGG1 RNAi)	AP3 and CETCH integrated (PLGG1 RNAi) *
LIGHT1_PSII	0,0439	0,0422	0,0353	0,0343	0,0776	0,0693	0,0725	0,0648
LIGHT2_CYTB6F	0,0877	0,0844	0,0706	0,0686	0,1552	0,1387	0,1451	0,1296
LIGHT3_PSI	0,1754	0,1687	0,1412	0,1372	0,3105	0,2774	0,2902	0,2593
LIGHT4_FNR	0,0877	0,0844	0,0706	0,0686	0,1552	0,1387	0,1451	0,1296
LIGHT5_ATPase	0,0439	0,0422	0,0353	0,0343	0,0776	0,0693	0,0725	0,0648
CBB1_RuBisCO1	0,1316	0,1265	0,1059	0,1029	0,2328	0,208	0,2176	0,1945
CBB2_PGK	0,0439	0,0422	0,0353	0,0343	0,0776	0,0693	0,0725	0,0648
CBB3_GAPDH	0,0439	0,0422	0,0353	0,0343	0,0776	0,0693	0,0725	0,0648
CBB4_FBPaldo	0,1316	0,1265	0,1412	0,1372	0,1552	0,1387	0,1451	0,1296
CBB5_FBPase	0,1316	0,1265	0,1412	0,1372	0,1552	0,1387	0,1451	0,1296
CBB6_TKTL1	0,1316	0,1265	0,1412	0,1372	0,1552	0,1387	0,1451	0,1296
CBB7_FBPaldo2	0,1316	0,1265	0,1412	0,1372	0,1552	0,1387	0,1451	0,1296
CBB8_SBPase	0,1316	0,1265	0,1412	0,1372	0,1552	0,1387	0,1451	0,1296
CBB9_TKTL2	0,1316	0,1265	0,1412	0,1372	0,1552	0,1387	0,1451	0,1296
CBB10_RPIA	0,1316	0,1265	0,1412	0,1372	0,1552	0,1387	0,1451	0,1296
CBB11_PRK	0,3947	0,3796	0,4237	0,4116	0,4657	0,416	0,4353	0,3889
CBB12_RPE	0,2631	0,2531	0,2825	0,2744	0,3105	0,2774	0,2902	0,2593
CBB13_TPI	0,2631	0,2531	0,2825	0,2744	0,3105	0,2774	0,2902	0,2593
PR1_RuBisCO2	0,2631	0,2531	0,3178	0,3087	0,2328	0,208	0,2176	0,1945
PR2_PGP	0,2631	0,2531	0,3178	0,3087	0,2328	0,208	0,2176	0,1945
TR1_PLGG1	0,2631	0,2531	0,2119	0,2058	--	--	--	--
PR3_GO	0,2631	0,2531	0,2119	0,2058	0	0	0	0
PR4_CAT1	0,0439	0,0422	0,0353	0,0343	0,0776	0,0693	0,0725	0,0648
PR5_GGT	0,1316	0,1265	0,1059	0,1029	0	0	0	0
TR2_TR6	0,2631	0,2531	0,2119	0,2058	0	0	0	0
PR6_SHMT	0,1316	0,1265	0,1059	0,1029	0	0	0	0
PR7_GDC	0,1316	0,1265	0,1059	0,1029	0	0	0	0
TR3_TR10	0,1316	0,1265	0,1059	0,1029	0	0	0	0
PR8_AGXT	0,1316	0,1265	0,1059	0,1029	0	0	0	0
PR9_HPR	0,1316	0,1265	0,1059	0,1029	0	0	0	0
TR4_PLGG1	0,1316	0,1265	0,1059	0,1029	--	--	--	--
PR10_GLYK	0,1316	0,1265	0,1059	0,1029	0	0	0	0

AP3_CrGDH	--	--	0,1059	0,1029	--	--	0,2176	0,1945
AP3_MS	--	--	0,1412	0,1715	--	--	0,2902	0,3241
CETCH1_CCR	0,0439	0,0844	0,0353	0,0686	0,0776	0,1387	0,0725	0,1296
CETCH2_CCR2	0,0439	0,0844	0,0353	0,0686	0,0776	0,1387	0,0725	0,1296
CETCH3_EPI-ECM	0,0439	0,0844	0,0353	0,0686	0,0776	0,1387	0,0725	0,1296
CETCH4_EPI-MCM	0,0439	0,0844	0,0353	0,0686	0,0776	0,1387	0,0725	0,1296
CETCH5_HBD	0,0439	0,0844	0,0353	0,0686	0,0776	0,1387	0,0725	0,1296
CETCH6_HBS	0,0439	0,0844	0,0353	0,0686	0,0776	0,1387	0,0725	0,1296
CETCH7_KAT	0,0439	0,0844	0,0353	0,0686	0,0776	0,1387	0,0725	0,1296
CETCH8_MCH	0,0439	0,0844	0,0353	0,0686	0,0776	0,1387	0,0725	0,1296
CETCH9_MCL	0,0439	0,0844	0,0353	0,0686	0,0776	0,1387	0,0725	0,1296
CETCH10_MCO	0,0439	0,0844	0,0353	0,0686	0,0776	0,1387	0,0725	0,1296
CETCH11_PCO	0,0439	0,0844	0,0353	0,0686	0,0776	0,1387	0,0725	0,1296
CETCH12_SCR	0,0439	0,0844	0,0353	0,0686	0,0776	0,1387	0,0725	0,1296
CETCH13_SSR	0,0439	0,0844	0,0353	0,0686	0,0776	0,1387	0,0725	0,1296
CETCH14_MAS	0,0439	0,0844	--	--	0,0776	0,1387	--	--

* Enoyl-CoA Carboxylases/Reductases (ECRs) are the most efficient CO2-fixing enzymes investigated in Schwander et al. and the crotonoyl-coA carboxylase/reductase (CCR) is one of the well known enzymes from this class (Schwander et al., 2016 : TableS1). Specifically for *M. extorquens* CCR used in the CETCH cycle, catalytic efficiency (kcat KM-1) is calculated to be between 5-7 x 105 M-1 s-1, which is approximately two- to four-fold more efficient then Rubisco (Peter, 2016 : Table 4.2). We represent this effect by increasing the activity of the EM spanning CETCH cycle by 2. This results in increased activities of CETCH cycle enzymes as well as increased AP3 MS activity and decreased photorespiration. The changes in enzyme activities represent the shift in flux distribution towards CETCH cycle.

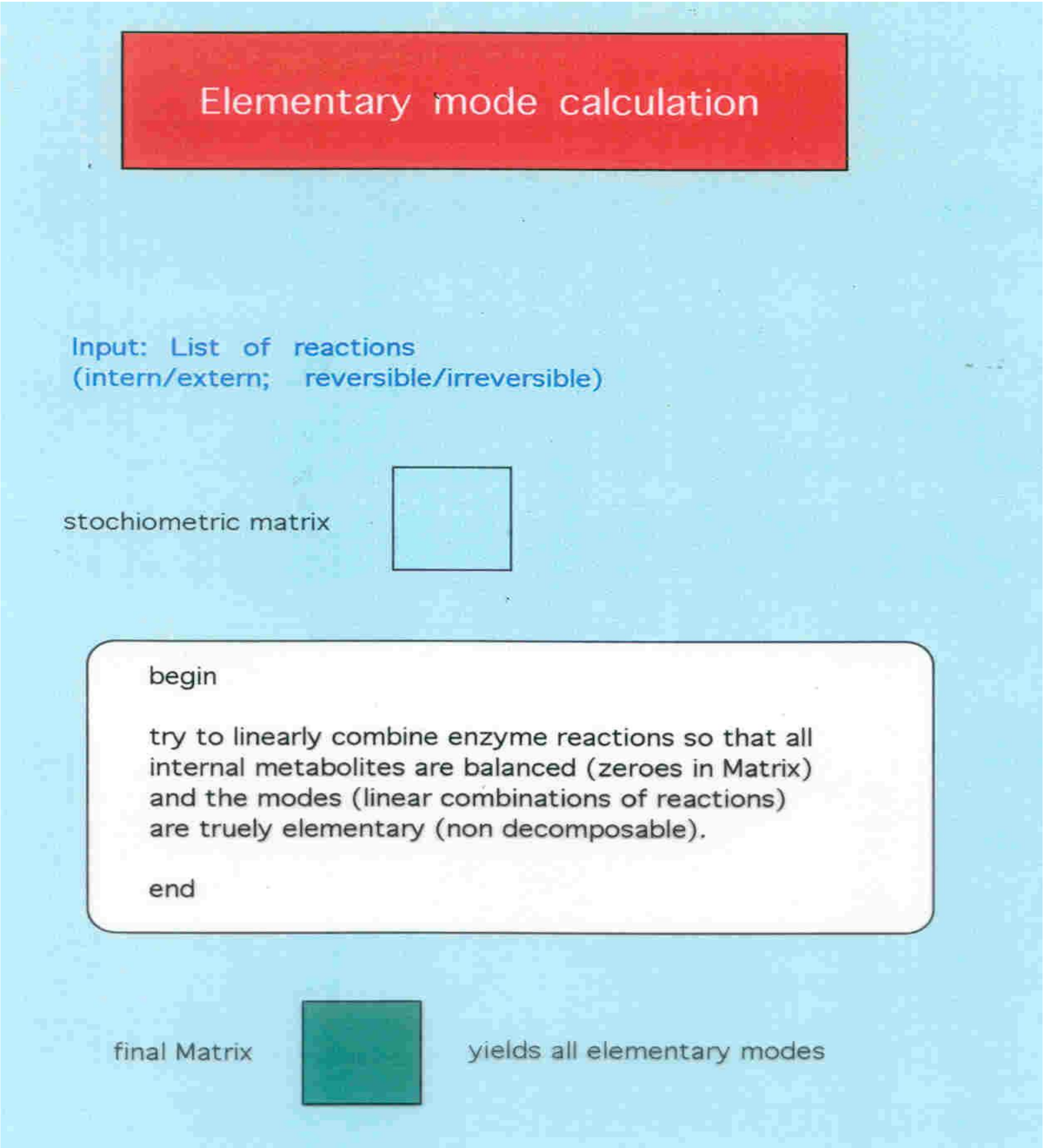

Figure S1. Flux balance analysis in a nutshell: enzyme combinations are found, that balance all involved internal metabolites within the network. This technique allows to enumerate all possible pathways and also identify pathway combinations which most efficiently fix carbondioxide from the environment.

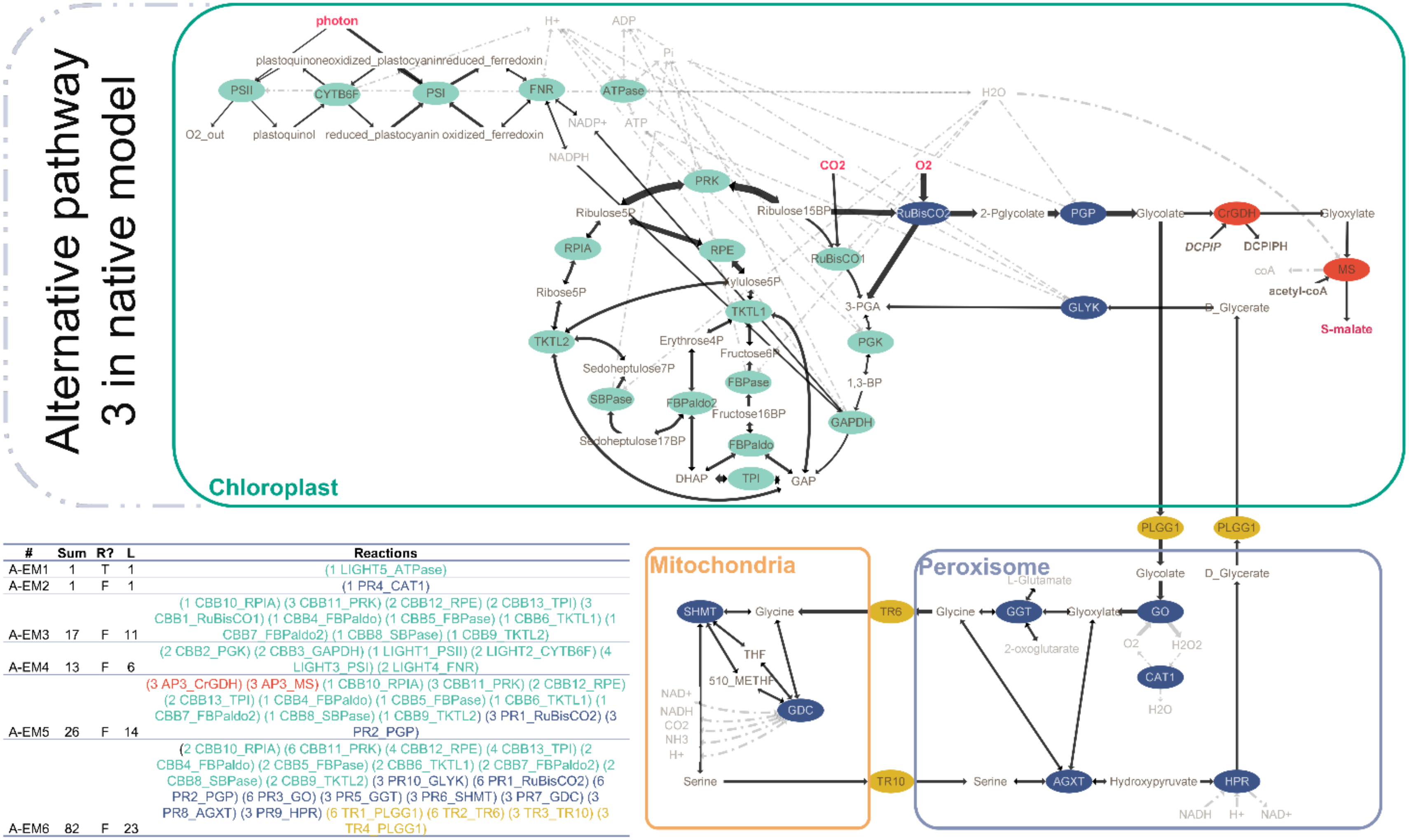

#	Sum	R?	L	Reactions
A-EM1	1	T	1	(1 LIGHT5_ATPase)
A-EM2	1	F	1	(1 PR4_CAT1)
A-EM3	17	F	11	(1 CBB10_RPIA) (3 CBB11_PRK) (2 CBB12_RPE) (2 CBB13_TPI) (3 CBB1_RuBisCO1) (1 CBB4_FBPaldo) (1 CBB5_FBPase) (1 CBB6_TKTL1) (1 CBB7_FBPaldo2) (1 CBB8_SBPase) (1 CBB9_TKTL2)
A-EM4	13	F	6	(2 CBB2_PGK) (2 CBB3_GAPDH) (1 LIGHT1_PSII) (2 LIGHT2_CYTB6F) (4 LIGHT3_PSI) (2 LIGHT4_FNR)
A-EM5	26	F	14	(3 AP3_CrGDH) (3 AP3_MS) (1 CBB10_RPIA) (3 CBB11_PRK) (2 CBB12_RPE) (2 CBB13_TPI) (1 CBB4_FBPaldo) (1 CBB5_FBPase) (1 CBB6_TKTL1) (1 CBB7_FBPaldo2) (1 CBB8_SBPase) (1 CBB9_TKTL2) (3 PR1_RuBisCO2) (3 PR2_PGP)
A-EM6	82	F	23	(2 CBB10_RPIA) (6 CBB11_PRK) (4 CBB12_RPE) (4 CBB13_TPI) (2 CBB4_FBPaldo) (2 CBB5_FBPase) (2 CBB6_TKTL1) (2 CBB7_FBPaldo2) (2 CBB8_SBPase) (2 CBB9_TKTL2) (3 PR10_GLYK) (6 PR1_RuBisCO2) (6 PR2_PGP) (6 PR3_GO) (3 PR5_GGT) (3 PR6_SHMT) (3 PR7_GDC) (3 PR8_AGXT) (3 PR9_HPR) (6 TR1_PLGG1) (6 TR2_TR6) (3 TR3_TR10) (3 TR4_PLGG1)

Figure S2. Alternative pathway 3 from South et al. (2019) integrated to the native model. Enzymes of photosynthesis are depicted in green, photorespiration in blue with transporters between organelles in gold, and AP3 in red. Reversible reactions shown with double-sided arrows.

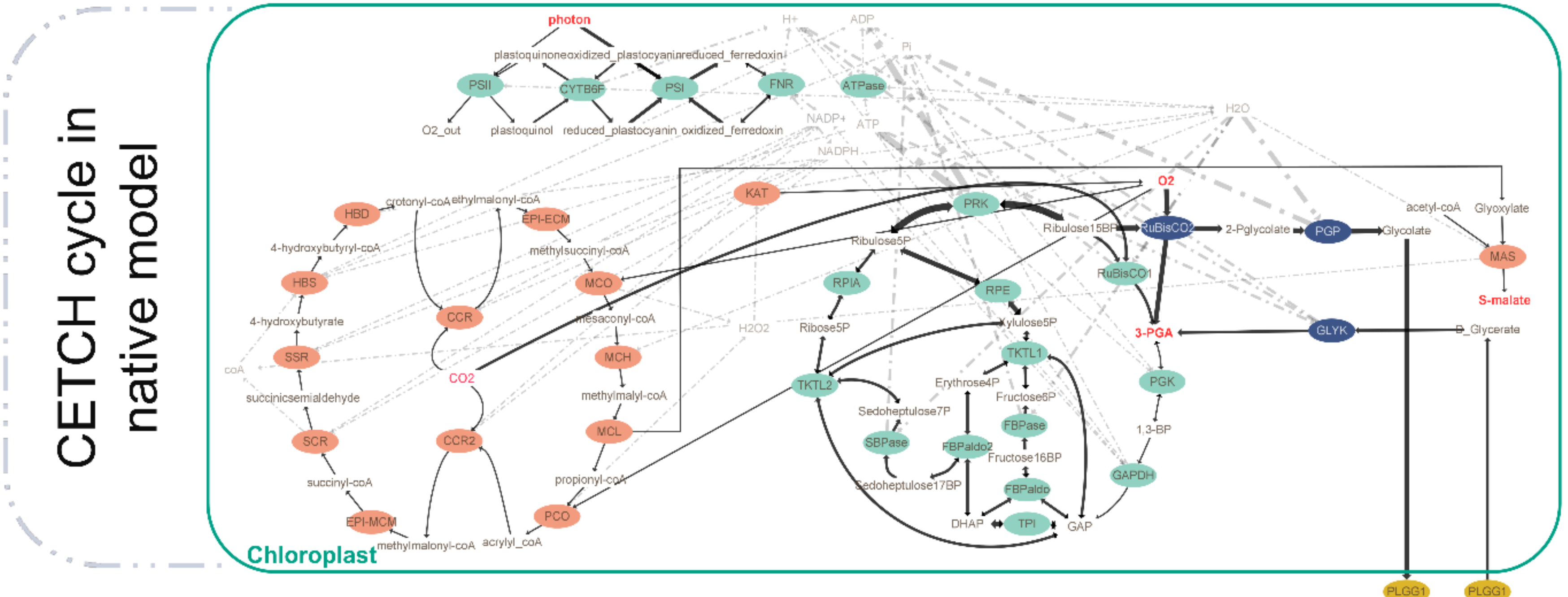

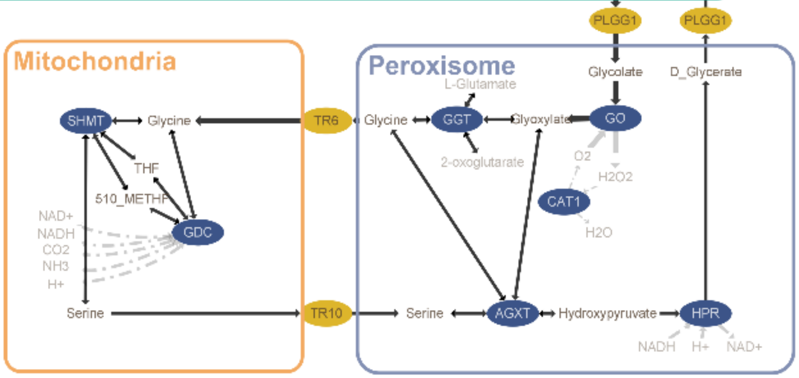

#	Sum	R?	L	Reactions
C-EM1	1	T	1	(1 LIGHT5_ATPase)
C-EM2	2	F	2	(1 CBB2_PGK) (1 CBB3_GAPDH)
C-EM3	1	F	1	(1 PR4_CAT1)
C-EM4	17	F	11	(1 CBB10_RPIA) (3 CBB11_PRK) (2 CBB12_RPE) (2 CBB13_TPI) (3 CBB1_RuBisCO1) (1 CBB4_FBPaldo) (1 CBB5_FBPase) (1 CBB6_TKTL1) (1 CBB7_FBPaldo2) (1 CBB8_SBPase) (1 CBB9_TKTL2)
C-EM5	9	F	4	(1 LIGHT1_PSII) (2 LIGHT2_CYTB6F) (4 LIGHT3_PSI) (2 LIGHT4_FNR)
C-EM6	82	F	23	(2 CBB10_RPIA) (6 CBB11_PRK) (4 CBB12_RPE) (4 CBB13_TPI) (2 CBB4_FBPaldo) (2 CBB5_FBPase) (2 CBB6_TKTL1) (2 CBB7_FBPaldo2) (2 CBB8_SBPase) (2 CBB9_TKTL2) (3 PR10_GLYK) (6 PR1_RuBisCO2) (6 PR2_PGP) (6 PR3_GO) (3 PR5_GGT) (3 PR6_SHMT) (3 PR7_GDC) (3 PR8_AGXT) (3 PR9_HPR) (6 TR1_PLGG1) (6 TR2_TR6) (3 TR3_TR10) (3 TR4_PLGG1)
C-EM7	14	F	14	(1 CETCH10_MCO) (1 CETCH11_PCO) (1 CETCH12_SCR) (1 CETCH13_SSR) (CETCH14_MAS) (1 CETCH1_CCR) (1 CETCH2_CCR2) (1 CETCH3_EPI-ECM) (1 CETCH4_EPI-MCM) (1 CETCH5_HBD) (1 CETCH6_HBS) (1 CETCH7_KAT) (1 CETCH8_MCH) (1 CETCH9_MCL)

Figure S3. CETCH cycle from Schwander et al. (2016) integrated to the native model. Enzymes of photosynthesis are depicted in green, photorespiration in blue with transporters between organelles in gold, and CETCH in orange. Reversible reactions shown with double-sided arrows.

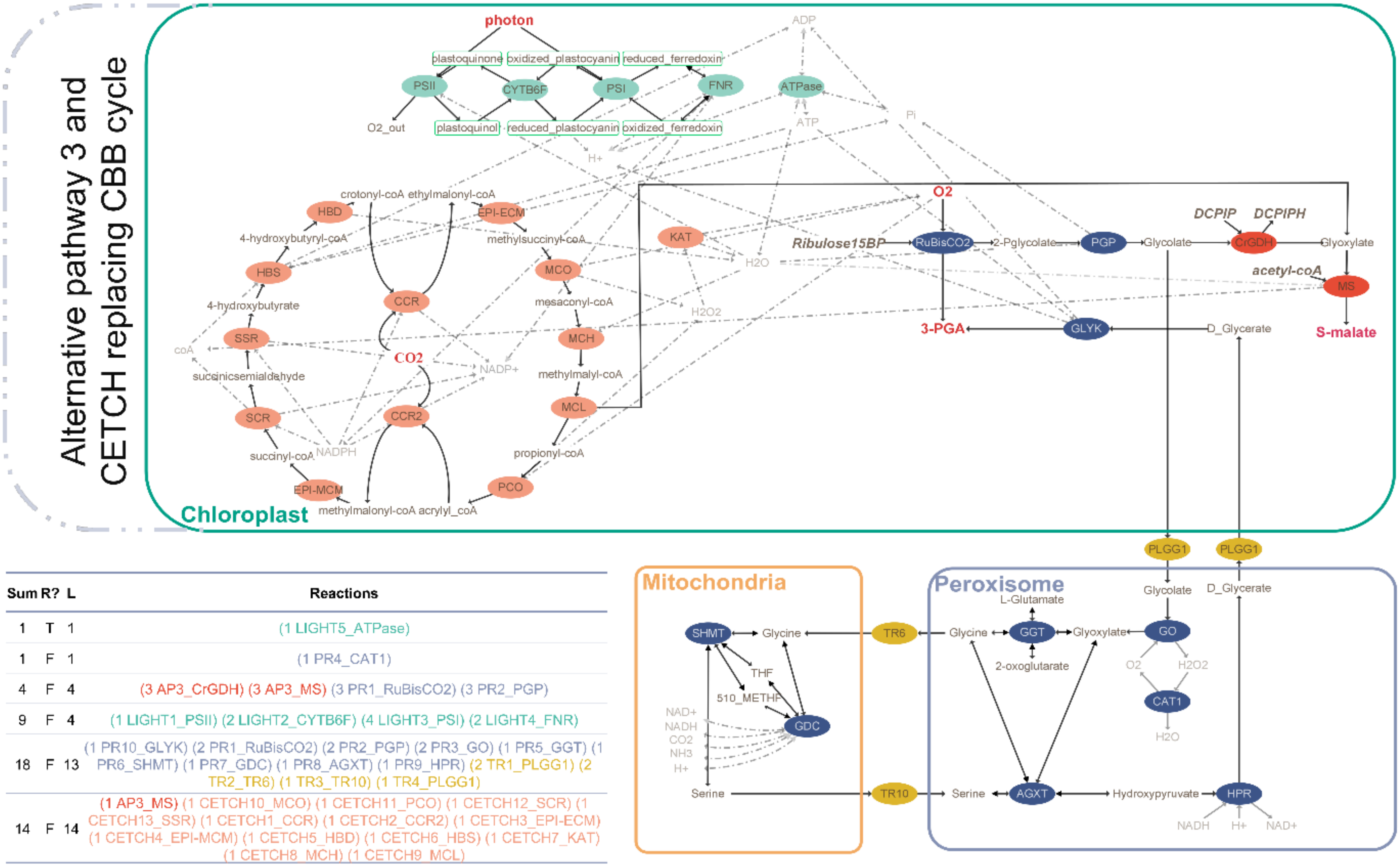

Sum	R?	L	Reactions
1	T	1	(1 LIGHT5_ATPase)
1	F	1	(1 PR4_CAT1)
4	F	4	(3 AP3_CrGDH) (3 AP3_MS) (3 PR1_RuBisCO2) (3 PR2_PGP)
9	F	4	(1 LIGHT1_PSII) (2 LIGHT2_CYTB6F) (4 LIGHT3_PSI) (2 LIGHT4_FNR)
18	F	13	(1 PR10_GLYK) (2 PR1_RuBisCO2) (2 PR2_PGP) (2 PR3_GO) (1 PR5_GGT) (1 PR6_SHMT) (1 PR7_GDC) (1 PR8_AGXT) (1 PR9_HPR) (2 TR1_PLGG1) (2 TR2_TR6) (1 TR3_TR10) (1 TR4_PLGG1)
14	F	14	(1 AP3_MS) (1 CETCH10_MCO) (1 CETCH11_PCO) (1 CETCH12_SCR) (1 CETCH13_SSR) (1 CETCH1_CCR) (1 CETCH2_CCR2) (1 CETCH3_EPI-ECM) (1 CETCH4_EPI-MCM) (1 CETCH5_HBD) (1 CETCH6_HBS) (1 CETCH7_KAT) (1 CETCH8_MCH) (1 CETCH9_MCL)

Figure S4. CETCH cycle from Schwander et al. (2016) replaced CBB cycle and AP3 integrated to the model. Enzymes of photosynthesis are depicted in green, photorespiration in blue with transporters between organelles in gold, and CETCH in orange, AP3 in red. Reversible reactions shown with double-sided arrows.

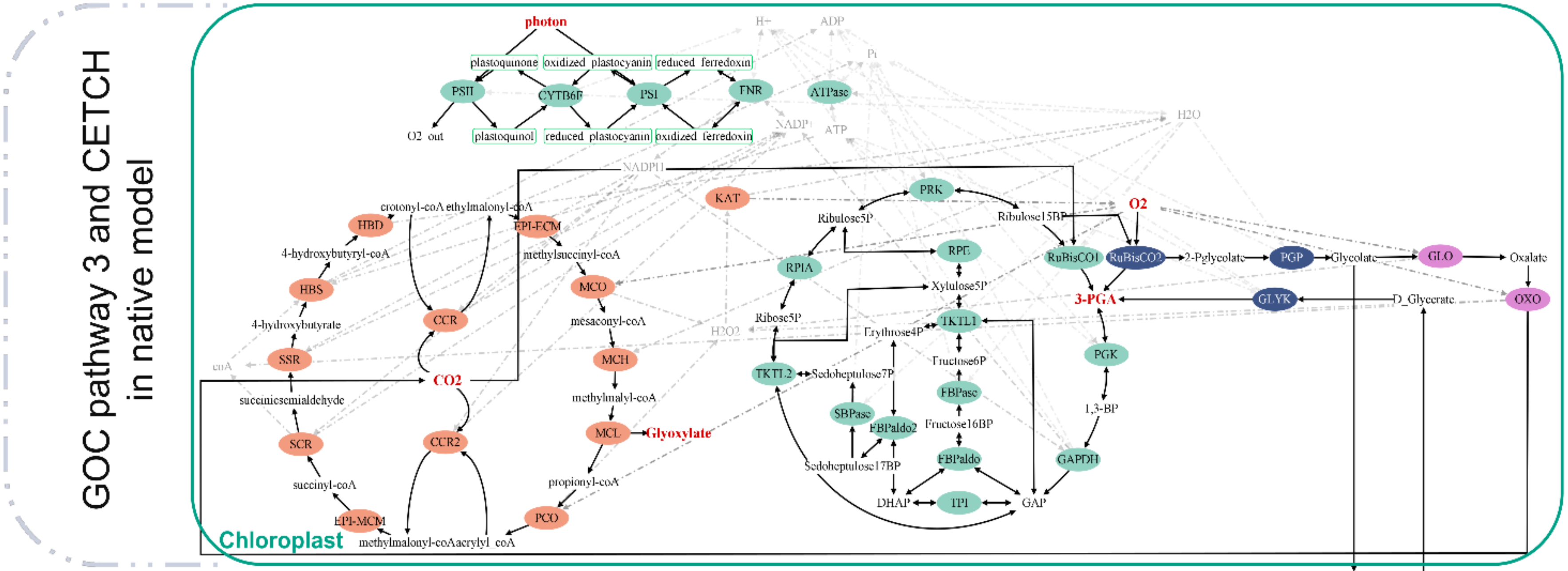

#	Sum	R?	L	Reactions
G-EM1	1	T	1	(1 LIGHT5_ATPase)
G-EM2	2	F	2	(1 CBB2_PGK) (1 CBB3_GAPDH)
G-EM3	1	F	1	(1 PR4_CAT1)
G-EM4	17	F	11	(1 CBB10_RPIA) (3 CBB11_PRK) (2 CBB12_RPE) (2 CBB13_TPI) (3 CBB1_RuBisCO1) (1 CBB4_FBPaldo) (1 CBB5_FBPase) (1 CBB6_TKTL1) (1 CBB7_FBPaldo2) (1 CBB8_SBPase) (1 CBB9_TKTL2)
G-EM5	26	F	14	(3 GOC_GLO) (3 GOC_OXO) (1 CBB10_RPIA) (3 CBB11_PRK) (2 CBB12_RPE) (2 CBB13_TPI) (1 CBB4_FBPaldo) (1 CBB5_FBPase) (1 CBB6_TKTL1) (1 CBB7_FBPaldo2) (1 CBB8_SBPase) (1 CBB9_TKTL2) (3 PR1_RuBisCO2) (3 PR2_PGP)
G-EM6	9	F	4	(1 LIGHT1_PSII) (2 LIGHT2_CYTB6F) (4 LIGHT3_PSI) (2 LIGHT4_FNR)
G-EM7	82	F	23	(2 CBB10_RPIA) (6 CBB11_PRK) (4 CBB12_RPE) (4 CBB13_TPI) (2 CBB4_FBPaldo) (2 CBB5_FBPase) (2 CBB6_TKTL1) (2 CBB7_FBPaldo2) (2 CBB8_SBPase) (2 CBB9_TKTL2) (3 PR10_GLYK) (6 PR1_RuBisCO2) (6 PR2_PGP) (6 PR3_GO) (3 PR5_GGT) (3 PR6_SHMT) (3 PR7_GDC) (3 PR8_AGXT) (3 PR9_HPR) (6 TR1_PLGG1) (6 TR2_TR6) (3 TR3_TR10) (3 TR4_PLGG1)
G-EM8	13	F	13	(1 CETCH10_MCO) (1 CETCH11_PCO) (1 CETCH12_SCR) (1 CETCH13_SSR) (1 CETCH1_CCR) (1 CETCH2_CCR2) (1 CETCH3_EPI-ECM) (1 CETCH4_EPI-MCM) (1 CETCH5_HBD) (1 CETCH6_HBS) (1 CETCH7_KAT) (1 CETCH8_MCH) (1 CETCH9_MCL)

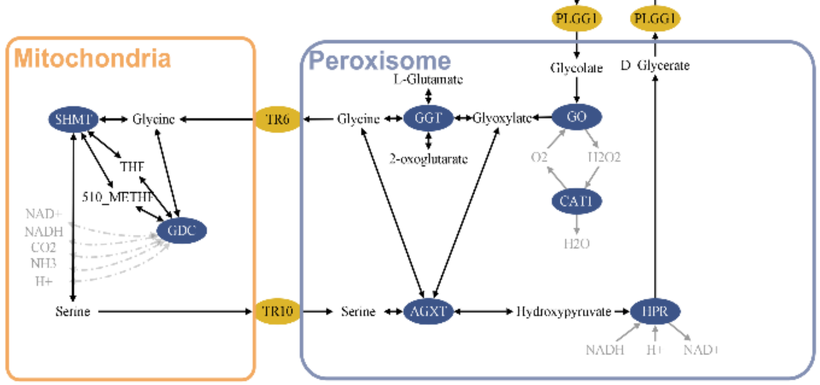

Figure S5. CETCH cycle from Schwander et al. (2016) integrated CBB cycle and GOC pathway from Bar-Even et al. (2010) (pink) integrated to the model**.** Enzymes of photosynthesis are depicted in green, photorespiration in blue with transporters between organelles in gold, and CETCH in orange. Reversible reactions shown with double-sided arrows.

References

Aboelmy, M.H., and Peterhansel, C. (2014). Enzymatic characterization of Chlamydomonas reinhardtii glycolate dehydrogenase and its nearest proteobacterial homologue. *Plant Physiol Biochem* 79, 25-30.

Carvalho Jde, F., Madgwick, P.J., Powers, S.J., Keys, A.J., Lea, P.J., and Parry, M.A. (2011). An engineered pathway for glyoxylate metabolism in tobacco plants aimed to avoid the release of ammonia in photorespiration. *BMC Biotechnol* 11, 111.

Cecil, A., Ohlsen, K., Menzel, T., Francois, P., Schrenzel, J., Fischer, A., Dorries, K., Selle, M., Lalk, M., Hantzschmann, J., Dittrich, M., Liang, C., Bernhardt, J., Olschlager, T.A., Bringmann, G., Bruhn, H., Unger, M., Ponte-Sucre, A., Lehmann, L., and Dandekar, T. (2015). Modelling antibiotic and cytotoxic isoquinoline effects in Staphylococcus aureus, Staphylococcus epidermidis and mammalian cells. *Int J Med Microbiol* 305, 96-109.

Kebeish, R., Niessen, M., Thiruveedhi, K., Bari, R., Hirsch, H.J., Rosenkranz, R., Stabler, N., Schonfeld, B., Kreuzaler, F., and Peterhansel, C. (2007). Chloroplastic photorespiratory bypass increases photosynthesis and biomass production in Arabidopsis thaliana. *Nat Biotechnol* 25, 593-599.

Löwe, H., and Kremling, A. (2021). In-depth computational analysis of natural and artificial carbon fixation pathways. *bioRxiv*, 2021.2001.2005.425423.

Maier, A., Fahnenstich, H., Von Caemmerer, S., Engqvist, M.K., Weber, A.P., Flugge, U.I., and Maurino, V.G. (2012). Transgenic Introduction of a Glycolate Oxidative Cycle into A. thaliana Chloroplasts Leads to Growth Improvement. *Front Plant Sci* 3, 38.

Peter, D.M. (2016). *Substrate Promiscuity, Kinetics and Engineering of Enoyl-CoA Carboxylases/Reductases*. Doctoral thesis, ETH Zürich.

Peterhansel, C., Blume, C., and Offermann, S. (2013). Photorespiratory bypasses: how can they work? *J Exp Bot* 64, 709-715.

Peterhansel, C., Horst, I., Niessen, M., Blume, C., Kebeish, R., Kurkcuoglu, S., and Kreuzaler, F. (2010). Photorespiration. *Arabidopsis Book* 8, e0130.

Schwander, T., Schada Von Borzyskowski, L., Burgener, S., Cortina, N.S., and Erb, T.J. (2016). A synthetic pathway for the fixation of carbon dioxide in vitro. *Science* 354, 900-904.

Schwarz, R., Liang, C., Kaleta, C., Kuhnel, M., Hoffmann, E., Kuznetsov, S., Hecker, M., Griffiths, G., Schuster, S., and Dandekar, T. (2007). Integrated network reconstruction, visualization and analysis using YANAsquare. *BMC Bioinformatics* 8, 313.

Schwarz, R., Musch, P., Von Kamp, A., Engels, B., Schirmer, H., Schuster, S., and Dandekar, T. (2005). YANA - a software tool for analyzing flux modes, gene-expression and enzyme activities. *BMC Bioinformatics* 6, 135.

Shen, B.R., Wang, L.M., Lin, X.L., Yao, Z., Xu, H.W., Zhu, C.H., Teng, H.Y., Cui, L.L., Liu, E.E., Zhang, J.J., He, Z.H., and Peng, X.X. (2019). Engineering a New Chloroplastic Photorespiratory Bypass to Increase Photosynthetic Efficiency and Productivity in Rice. *Mol Plant* 12, 199-214.

South, P.F., Cavanagh, A.P., Liu, H.W., and Ort, D.R. (2019). Synthetic glycolate metabolism pathways stimulate crop growth and productivity in the field. *Science* 363.

Xin, C.P., Tholen, D., Devloo, V., and Zhu, X.G. (2015). The benefits of photorespiratory bypasses: how can they work? *Plant Physiol* 167**,** 574-585.
